# Immunomodulating Therapies in Acute Myocarditis and Recurrent/Acute Pericarditis

**DOI:** 10.3389/fmed.2022.838564

**Published:** 2022-03-07

**Authors:** Enrico Ammirati, Emanuele Bizzi, Giacomo Veronese, Matthieu Groh, Caroline M. Van de Heyning, Jukka Lehtonen, Marc Pineton de Chambrun, Alberto Cereda, Chiara Picchi, Lucia Trotta, Javid J. Moslehi, Antonio Brucato

**Affiliations:** ^1^De Gasperis Cardio Center and Transplant Center, Niguarda Hospital, Milano, Italy; ^2^Internal Medicine, Fatebenefratelli Hospital, Milano, Italy; ^3^Department of Health Sciences, University of Milano-Bicocca, Monza, Italy; ^4^National Reference Center for Hypereosinophilic Syndromes, CEREO, Suresnes, France; ^5^Department of Internal Medicine, Hôpital Foch, Suresnes, France; ^6^Department of Cardiology, Antwerp University Hospital, and GENCOR Research Group, Antwerp University, Antwerp, Belgium; ^7^Department of Cardiology, Heart and Lung Center, Helsinki University Hospital, Helsinki, Finland; ^8^Sorbonne Université, Assistance Publique-Hôpitaux de Paris (APHP), Hôpital La Pitié-Salpêtrière, Service de Médecine Intensive-Réanimation, Paris, France; ^9^Sorbonne Université, APHP, Hôpital de la Pitié-Salpêtrière, Service de Médecine Interne 2, Centre de Référence National Lupus et SAPL et Autres Maladies Auto-immunes et Systémiques Rares, Paris, France; ^10^Sorbonne Université, INSERM, UMRS_1166-ICAN, ICAN, Paris, France; ^11^Cardiovascular Department, Association Socio Sanitary Territorial Santi Paolo e Carlo, Milano, Italy; ^12^Section of Cardio-Oncology and Immunology, Division of Cardiology and the Cardiovascular Research Institute, University of California, San Francisco, San Francisco, CA, United States; ^13^Department of Biomedical and Clinical Sciences “Luigi Sacco, ” Fatebenefratelli Hospital, University of Milano, Milano, Italy

**Keywords:** acute myocarditis, pericarditis, immunosuppressive therapy, eosinophilic myocarditis, COVID-19, cardiac sarcoidosis, corticosteroids, anti-IL-1 therapy

## Abstract

The field of inflammatory disease of the heart or “cardio-immunology” is rapidly evolving due to the wider use of non-invasive diagnostic tools able to detect and monitor myocardial inflammation. In acute myocarditis, recent data on the use of immunomodulating therapies have been reported both in the setting of systemic autoimmune disorders and in the setting of isolated forms, especially in patients with specific histology (e.g., eosinophilic myocarditis) or with an arrhythmicburden. A role for immunosuppressive therapies has been also shown in severe cases of coronavirus disease 2019 (COVID-19), a condition that can be associated with cardiac injury and acute myocarditis. Furthermore, ongoing clinical trials are assessing the role of high dosage methylprednisolone in the context of acute myocarditis complicated by heart failure or fulminant presentation or the role of anakinra to treat patients with acute myocarditis excluding patients with hemodynamically unstable conditions. In addition, the explosion of immune-mediated therapies in oncology has introduced new pathophysiological entities, such as immune-checkpoint inhibitor-associated myocarditis and new basic research models to understand the interaction between the cardiac and immune systems. Here we provide a broad overview of evolving areas in cardio-immunology. We summarize the use of new imaging tools in combination with endomyocardial biopsy and laboratory parameters such as high sensitivity troponin to monitor the response to immunomodulating therapies based on recent evidence and clinical experience. Concerning pericarditis, the normal composition of pericardial fluid has been recently elucidated, allowing to assess the actual presence of inflammation; indeed, normal pericardial fluid is rich in nucleated cells, protein, albumin, LDH, at levels consistent with inflammatory exudates in other biological fluids. Importantly, recent findings showed how innate immunity plays a pivotal role in the pathogenesis of recurrent pericarditis with raised C-reactive protein, with inflammasome and IL-1 overproduction as drivers for systemic inflammatory response. In the era of tailored medicine, anti-IL-1 agents such as anakinra and rilonacept have been demonstrated highly effective in patients with recurrent pericarditis associated with an inflammatory phenotype.

## Introduction

The field of inflammatory disease of the heart or “cardio-Immunology” is rapidly evolving thanks to the wider use of non-invasive diagnostic tools able to detect and monitor myocardial inflammation, such as cardiac magnetic resonance imaging (CMRI) and fluorodeoxyglucose positron emission tomography (FDG-PET) (1). In acute myocarditis (AM), recent data on the use of immunomodulating therapies have been reported both in the setting of systemic autoimmune disorders and in the setting of isolated forms, especially in patients with specific histology (i.e., eosinophilic myocarditis, giant cell myocarditis [GCM] or cardiac sarcoidosis [CS]) or characterized by an arrhythmic burden (2). We elucidate the rationale to test the use of immunomodulating therapies in patients with lymphocytic AM. In addition, AM has also emerged as a complication in the setting of coronavirus disease 2019 (COVID-19), mRNA vaccine (3–7), and immune checkpoint inhibitors (ICI) (8–10). Here, we summarize the clinical approach toward the use of immunosuppressive therapies in these specific settings. Finally, we propose the use of new imaging tools in combination with endomyocardial biopsy (EMB) and laboratory parameters such as high sensitivity troponin to monitor the response to immunomodulating therapies based on recent evidence and clinical experience.

In the second section of this review, we examine the rationale and the evidence of immunosuppression in pericarditis. We highlight recent findings defining a pivotal role for innate immunity in the pathogenesis of recurrent pericarditis with raised C-reactive protein (CRP), focusing on the emerging role of anti-IL-1 agents (i.e., anakinra and rilonacept) for this subset of patients with recurrent pericarditis.

## Lymphocytic Myocarditis

**Figure 1:**
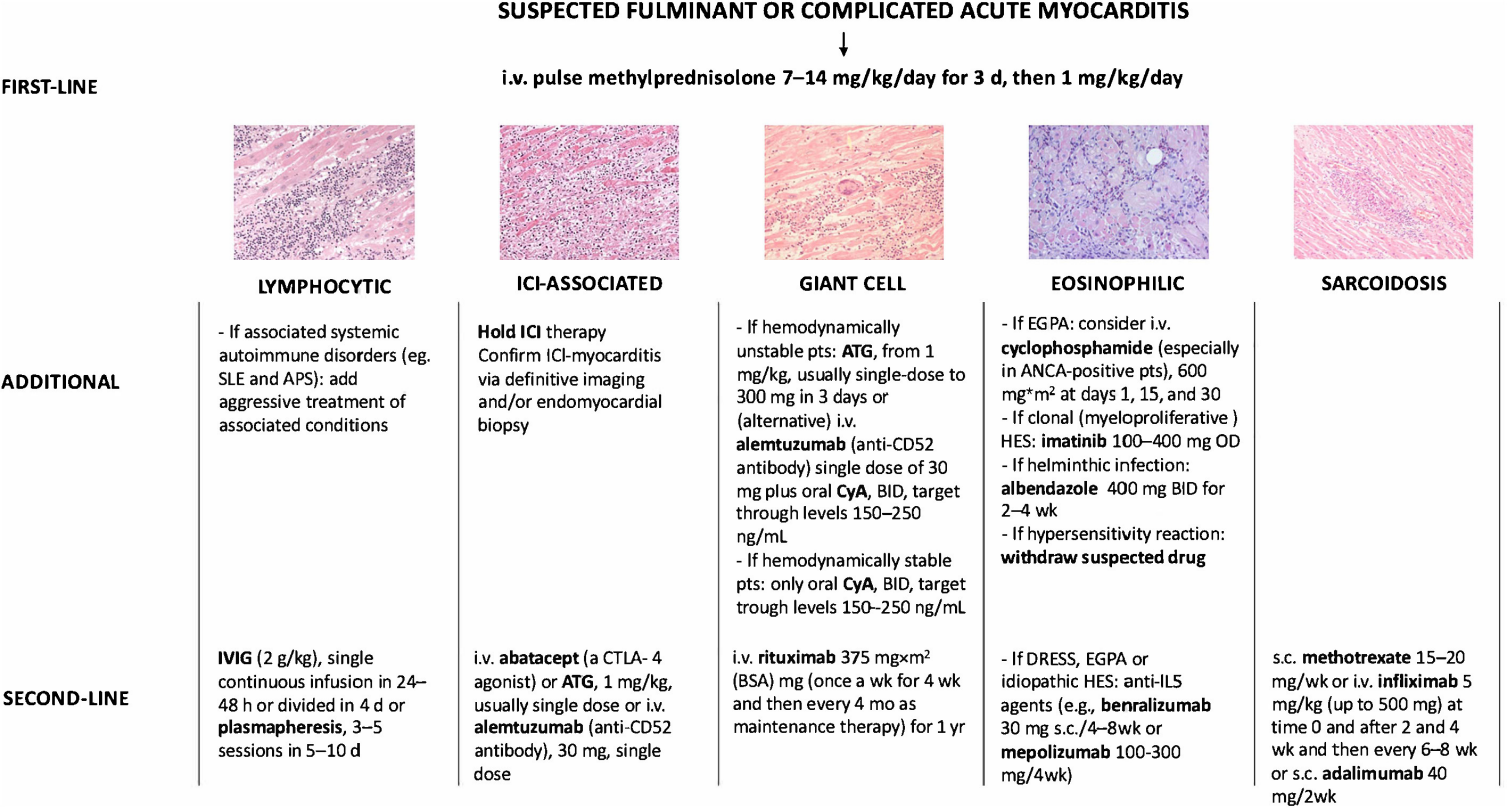
Immunosuppressive treatment strategies used for fulminant myocarditis or complicated acute myocarditis not supported by evidence from clinical trials but based on published case reports/series. i.v., intravenous; d, day; IVIG, intravenous immunoglobulin; h, hour; SLE, systemic lupus erythematosus; APS, antiphospholipid syndrome; ICI, immune checkpoint inhibitor; pts, patients; ATG, anti-thymocyte globulin; wk, week; CyA, cyclosporine; mo, month; EGPA, eosinophilic granulomatosis with polyangiitis; ANCA, antineutrophil cytoplasmatic antibodies; DRESS, drug reaction with eosinophilia asn dystemic symptoms; HES, hypereosinophilic syndrome; s.c., subcutaneous. Adapted from Ammirati et al. (1).

Lymphocytic AM is the most common histologic subset reported in AM cohorts (11). Due to the fact that in the setting of suspected AM, histologic diagnosis is more often recommended in specific scenarios (e.g., acute heart failure [HF], presence of ventricular arrhythmias (VA) or II/III-degree atrio-ventricular block [AVB]) (1, 12), the prevalence of lymphocytic AM is frequently estimated on cohorts of complicated AM. From a recent international retrospective case collection of AM presenting with left ventricular (LV) systolic dysfunction, the prevalence of lymphocytic AM has been estimated to be ~72%, being the most frequently diagnosed form both in fulminant myocarditis [FM], a clinical entity defined by the need of circulatory support, and non-FM (11). The etiology of lymphocytic AM is broad and includes heterogeneous pathogens, drugs or autoimmune-mediated injury in the setting of systemic inflammatory diseases (10, 13, 14). The role of viruses in myocarditis etiology has been historically recognized, with Parvovirus (PV)B-19, adenoviruses, Human Herpesvirus (HHV)-6, enteroviruses being the most common agents identified in the myocardium of patients with AM (15, 16). Whether viruses have a direct or indirect causal relationship in clinical myocarditis etiology has been a matter of great debate throughout the years with expert opinions varying according to the evidence of the moment (17). The controversy matters as it has been stated that the presence of specific viruses in the heart may be a contraindication to the use of immunosuppression (18). A growing body of literature indicates that viruses, particularly PVB-19 and HHV6, may be found in a large proportion of patients who do not have myocarditis, questioning their direct causal role in the pathogenesis of myocarditis (19, 20). Of note, PVB-19 was the only virus identified in patients with lymphocytic FM in an international registry (21). Except for enteroviruses (22, 23), such as coxsackievirus, whose ability to cause direct myocardial damage has been demonstrated and seems more common in newborns/infants (24), most of the available evidence suggests that virus-triggered immune-mediated reactions are the principal cause of cardiomyocyte injury (1). Respiratory viruses, such as influenza and coronaviruses, are examples of common viruses that can trigger immune-mediated lymphocytic myocarditis with no evidence of viral genome in the myocardium (25, 26). Molecular mimicry between viral and cardiac antigens is suspected to be a key mechanism of myocardial injury in virus-triggered AM (27, 28). Furthermore, the concept that FM may resemble the presentation of a high-grade cellular rejection observed after heart transplantation (HTx) is recently emerging. These findings may suggest that the identification of viruses in the setting of AM may not represent an absolute contraindication to immunosuppression (29). At present, the role of a routine viral genome search on EMB in guiding patient management and immunosuppression therapy in patients with AM remains unknown (17). This concept holds true especially in FM where early immunosuppression may be crucial to damper the inflammatory process sustaining AM. However, most studies focusing on immunomodulation have included patients with chronic inflammatory cardiomyopathy with HF symptoms for more than 6 months rather than those with a fulminant or complicated course (30–32). Though not supported by evidence from randomized clinical trials, recommendations for immunosuppression exist in the setting of complicated AM based on case series, expert opinions, and pathophysiological considerations (1) (Figure 1). The American Heart Association (AHA) suggests that, if a high suspicion for immune-mediated FM exists, pulse steroid therapy (i.e., 1 g of methylprednisolone) should be administered urgently, before biopsy-confirmed diagnosis or further diagnostic testing (33). Intravenous (IV) immunoglobulin (IG) (at a dose ranging from 0.5 g to 1 g/kg) is frequently used in pediatric lymphocytic myocarditis with evidence of some benefits in terms of functional recovery and survival, but the experience in adults has been limited (34, 35). Even though not standardized, maintenance therapy with low dose steroids often in combination with mycophenolate mofetil, cyclosporine, azathioprine (AZA) as steroid-sparing drugs may be used in those patients showing poor functional recovery associated with persistence of troponin release or any evidence of residual myocardial inflammation (30, 36). Standardized Corticosteroid therapy (IV methylprednisolone 200–400 mg or dexamethasone 20–40 mg) qd for 3–5 days and then gradually down titrated and weaned in 7–10 days, and IVIG 10–20 g qd for 3–5 days followed by 10 g for another 3–5 days has been described from a Chinese registry of 138 FM and has been associated with improved survival (37). According to several researchers, even though robust evidence is substantially lacking in the setting of AM, high viral loads may contraindicate the use of immunosuppression in favor of treatment with antiviral drugs or with agents boosting the native immune response (e.g., interferon-β) (38). Lymphocytic AM can also be associated with systemic autoimmune or inflammatory disorders (e.g., systemic lupus erythematosus [SLE], inflammatory bowel disorders, COVID-19) (39). The Lombardy registry of AM reported that 7.2% of patients had associated autoimmune or systemic disorders, being more frequent in patients presenting with complicated AM (40). The identification of the myocarditis-associated condition is essential to initiate disease-specific treatments. IV corticosteroids have been successfully used in cases of SARS-CoV-2 related FM, suggesting the relevance of the systemic inflammatory response in determining cardiac injury in COVID-19, even though more evidence is needed (41, 42).

### Ongoing Trials

**Figure 2:**
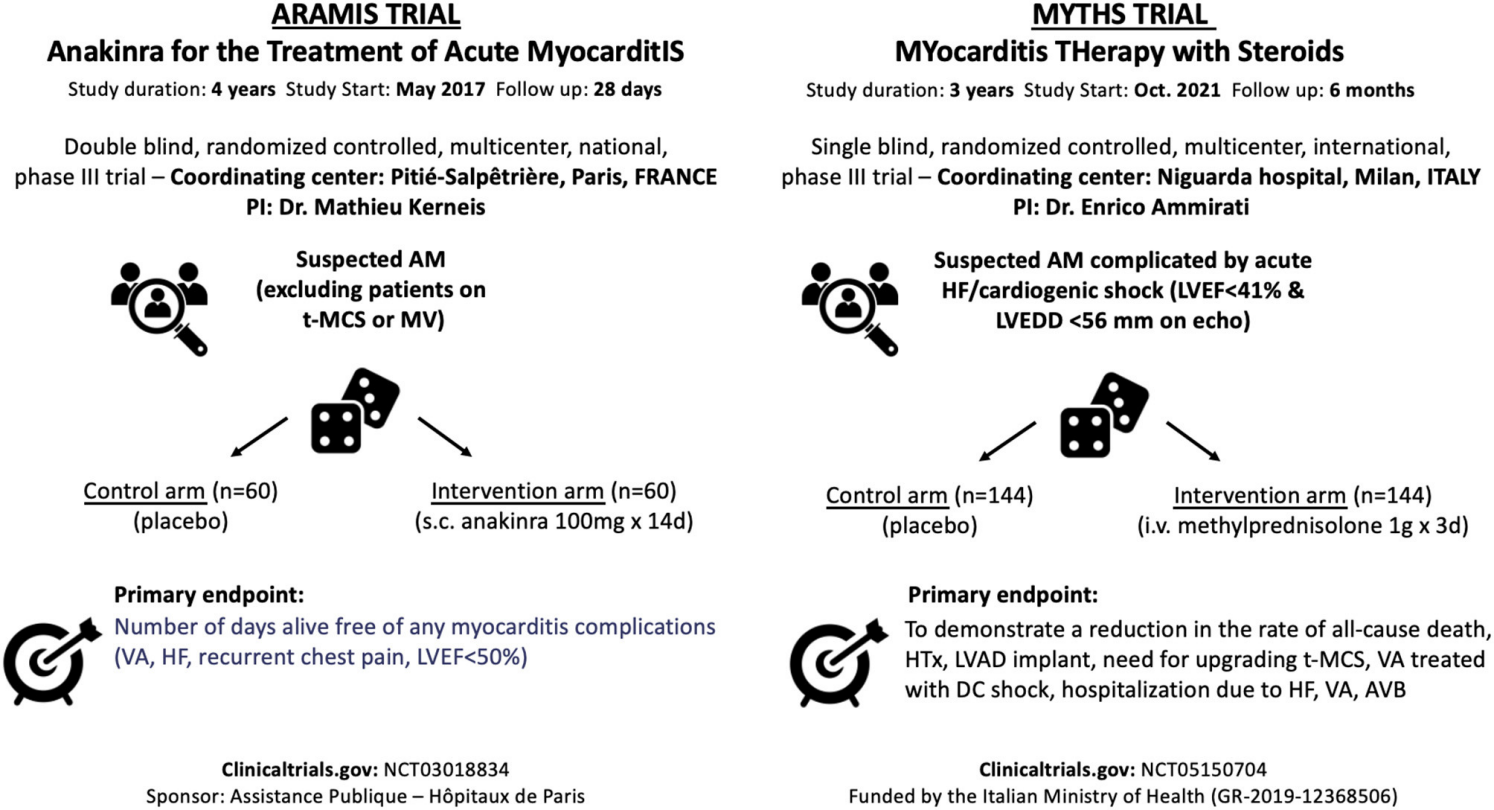
Ongoing trials in the setting of acute myocarditis evaluating the use of immunosuppressive drugs. PI, principal investigator; AM, acute myocarditis; HF, heart failure; LVEF, left ventricular ejection fraction; LVEDD, left ventricular end-diastolic diameter; iv, intravenous; d, days; HTx, heart transplant; LVAD, left ventricular assist device; t-MCS, temporary-mechanical circulatory support; VA, ventricular arrhythmias; AVB, atrioventricular block; MV, mechanical ventilation; sc, subcutaneous.

Anakinra is the recombinant form of the naturally occurring interleukin 1α (IL-1Rα) and blocks the activity of both IL-1α and IL-1β. The Anakinra vs. Placebo for the Treatment of Acute Myocarditis (ARAMIS) trial (ClinicalTrials.gov identifier: NCT03018834) is a double-blind randomized clinical trial testing the superiority of anakinra in addition to standard of care, defined as the maximum tolerated dosage of any beta-blockers and angiotensin receptor blockade in acute myocarditis. The ARAMIS trial has completed the randomization phase and will directly assess the role of the IL-1 immune innate pathway in the setting of AM. The rationale of blocking the (IL-1β) pathway in myocarditis relies on prior studies that suggested the central role of the Nucleotide-binding domain (NACHT) and Leucine-rich repeat (LRR) and Pyrin domain (PYD) (NLR) containing protein 3 (NLRP3) inflammasome predominately expressed in macrophages (43–45). Despite anecdotal evidence, ARAMIS will directly test this concept and the results are expected by the end of 2022 (46, 47). This double-blinded French study has assessed 120 patients with symptomatic AM defined by elevated cardiac troponin (at least 1.5-fold upper the normal reference limit) and CMRI consistent with myocarditis performed within 72 h after admission (Figure 2). Patients in the treatment arm received a daily subcutaneous dose of anakinra 100 mg during the hospitalization including an angiotensin-converting-enzyme inhibitor (ACE-i) and a beta-blocker. The primary endpoint of this study is the number of days alive free of any myocarditis complications including (1) VA, (2) HF, (3) recurrent chest pain requiring medication, (4) left ventricular ejection fraction (LVEF) <50%, up to 28 days after randomization. This trial has also a sub-study that has assessed ACE-i continuation or discontinuation after 1 month in patients with normal LVEF that are followed for 1 year. This trial excluded the patients with the poorest outcome, specifically those on mechanical ventilation or temporary mechanical circulatory supports (t-MCS). To address specifically patients with FM or acute HF the MYocarditis THerapy with Steroids (MYTHS) trial (ClinicalTrials.gov identifier: NCT05150704) will randomize 288 patients with FM (need for inotropes and/or t-MCS) or AM complicated by HF and severely impaired LVEF (<41%) to pulsed corticosteroid therapy (methylprednisolone 1 g IV qd for 3 days) on top of standard therapy and maximal supportive care vs. placebo (Figure 2). The combined primary endpoint is defined as the time from randomization to the first event occurring within 6 months including (1) all-cause death, or (2) HTx, or (3) long-term left-ventricular assistance device (LVAD) implant, or (4) need for an upgrading of the t-MCS, or (5) a ventricular tachycardia (VT)/fibrillation (VF) treated with direct current (DC) shock (excluding VT/VF in patients on t-MCS other than intra-aortic balloon pump [IABP]), or (6) first rehospitalization due to HF or VA, or advanced AVB. The trial started the enrollment in October 2021 and the estimated duration is ~3–4 years. The rationale for the MYTHS trial is based on clinical practice. Indeed, several case series and case reports support the effectiveness of high dosage corticosteroids (48–50).

## Specific Subset of Myocarditis

### Myocarditis in Systemic Lupus Erythematosus and Antiphospholipid Antibody Syndrome

SLE is a rare disease (prevalence 48–350 per 100,000 individuals) in which the immune system attacks healthy cells and tissues. Immune system activation is characterized by exaggerated B/T cell responses and loss of tolerance against self-antigens. Production and defective elimination of antibodies, tissue deposition of immune complexes, and complement and cytokine activation contribute to clinical manifestations ranging from joint and skin inflammation to life-threatening organ damage (51). Young women are disproportionately affected by SLE with a female-to-male sex ratio around 10:1 (52). Lupus myocarditis is a rare manifestation of SLE occurring in <5% of patients (53, 54) frequently at disease onset (≈60% of cases) (55). Cardiac manifestations of SLE-myocarditis are nonspecific: elevated troponin 80%, abnormal electrocardiogram 90%, altered LVEF (≤ 45%) 66%, pericardial effusion 69% (55), and usually associated with other SLE clinical features (e.g., fever, skin rash, joint inflammation, lupus nephritis). When isolated lupus myocarditis is suspected, SLE diagnosis relies on: positive anti-nuclear, anti-dsDNA (ELISA, Crithidia luciliae or Farr tests) or anti-extractable nuclear antigen (especially anti-SM) antibodies; low C3 complement fraction and/or elevated serum interferon-alpha (56, 57). CMRI usually reveals cardiac inflammation and the presence of late gadolinium enhancement (LGE) (69%) (55). EMB for the diagnosis of lupus myocarditis has shown disappointing results (58) and its use is debatable owing to the numerous non-invasive diagnosis methods of SLE, at least in the case of patients with stable hemodynamic conditions. Moreover, the histopathologic abnormalities of lupus myocarditis (lymphocytic myocarditis) are non-specific, even if SLE can be occasionally associated with GCM (59). The management of lupus myocarditis is not specifically addressed in the latest guidelines for the management of SLE (60). General consensus suggests the use of high-dose corticosteroids with the addition of an immunosuppressive drug (e.g., cyclophosphamide) in patients who are refractory to corticosteroids alone (Figure 1). Under these therapies, LVEF can recover to a normal value in most patients (>80%) (55).

Antiphospholipid antibody syndrome (APLAS) is a rare systemic autoimmune disease responsible for thrombotic events and obstetric morbidity in patients with persistent antiphospholipid autoantibodies (lupus anticoagulant, anticardiolipin and/or anti-beta2 glycoprotein [GP]-1 antibodies) (61). APLAS is the leading cause of acquired thrombophilia accounting for 10% of arterial or venous thrombosis. The disease mainly occurs in young adults (mean age at diagnosis 34–54 years) with a sex ratio slightly favoring women (55–82%) (62, 63) and can be associated with other autoimmune diseases, especially SLE. APLAS can induce chronic valvular lesions (Libman-Sacks endocarditis) responsible for mitral (more frequently) and/or aortic stenosis and/or regurgitation (64). Myocardial infarction in the setting of APLAS can be related to macrovascular thrombosis of coronary vessels or to microvascular thrombosis (myocardial infarction with non-obstructive coronary arteries [MINOCA]). The clinical features of APS-related MINOCAs are non-specific and associated with chest pain, electrocardiographic changes, a rise in cardiac necrosis markers, and evidence of myocardial LV systolic dysfunction. Macrovascular or microvascular thrombosis frequently occurs as *thrombotic storm* termed catastrophic APLAS (C-APLAS). The C-APLAS is defined as the occurrence of (1) at least the involvement of 3 organs, tissues, or systems in <7 days; (2) with biopsy-proven small vessel occlusion; (3) in patients with persistent high title of antiphospholipid antibodies (65). These classification criteria should be considered with great caution as they do not encompass the full spectrum of severe APLAS microvascular thrombotic episodes and some patients may require treatment escalation even though they do not fulfill the criteria for C-APLAS (66). When available, EMB can reveal myocyte necrosis with small vessels occlusions (67). However, EMB is generally not performed as it is perceived at increased risk of a bleeding complication. Small vessel occlusion can alternatively be disclosed on biopsy from other organs (i.e., skin) and CMRI can help identify microvascular occlusion (68). Nevertheless, in critically-ill patients EMB can differentiate scenario where inflammatory infiltrates prevails over the small vessels occlusions or it can reveal a GCM (1). The treatment of APS relies on anticoagulation as neither corticosteroids nor immunosuppressants nor biologics have proven their efficacy (69). Nevertheless, patients with C-APLAS should be given a triple therapy associating anticoagulation, high-dose corticosteroids, and either IVIG or plasma exchange (70). Rituximab has been also frequently used in combination with plasma exchange in C-APLAS with myocarditis (67, 71, 72). In refractory cases, the use of complement inhibitors (i.e., eculizumab) can be discussed on a case-by-case basis (73).

### Immune Checkpoint Inhibitor Associated Myocarditis

ICIs have transformed cancer treatment and include monoclonal antibodies which block immune brakes such as CTLA-4 (cytotoxic T-lymphocyte antigen-4), PD-1 (programmed death receptor-1), and its ligand (PD-L1 [programmed death-ligand 1]), leading to reinvigoration of T cell responses against cancer (74). By activating the immune system, ICI can also lead to immune-related adverse events (irAE) which can affect any organ (75, 76). Myocarditis is one of the most serious irAE associated with ICI use (77). Initially described in 2016, ICI-myocarditis is now considered an infrequent but potentially lethal complication of ICI (78). ICI-myocarditis is especially arrhythmogenic and is pathologically characterized by T-cell and macrophage infiltration of the myocardium (79). Systolic HF occurs in about half of patients. On the other hand, ICI-myocarditis often occurs concomitantly with myositis, as well as a myasthenia-like syndrome (80–82). The main risk factor is combination ICI treatment, for example, when ipilimumab (anti-CTLA-4) and nivolumab (anti-PD1) are combined for more effective treatment (9). Clinical definitions have been established and advocate for the use of biomarkers, imaging, and EMB for optimal and prompt diagnosis of treatment (83, 84). Preclinical models of ICI-myocarditis have been established and suggest a critical role for immune checkpoints in the heart. For example, genetic absence of *Pdcd1* (encoding PD-1) and *Ctla4* (encoding for CTLA-4) haploinsufficiency recapitulate various features of ICI-associated myocarditis, including myocardial infiltration by T cells and severe electrocardiographic abnormalities (i.e., sinus node dysfunction, sinus arrest, and atrioventricular conduction block) (85, 86). Therapeutic intervention with abatacept (recombinant CTLA-4 immunoglobulin) rescues the fatal myocarditis in this mouse model, providing mechanistic support for inhibition of T cell co-stimulation mediated by CTLA-4 as a treatment for ICI-associated myocarditis. Anecdotal evidence supports the use of abatacept in severe cases of ICI-myocarditis (87). ICI-induced myocarditis affects elder patients (median age of 65 years) with more comorbidities compared with non-ICI-induced myocarditis (median age between 30 and 40 years) (40, 88–90). One of the largest case series of 122 patients with ICI-associated myocarditis had early onset of symptoms (median 30 days after initial exposure to ICI), and up to 50% of deaths (9). A systematic analysis of the World Health Organization pharmacovigilance database confirmed a 32.5% of mortality in patients who had myocarditis associated with the administration of ICIs with a median time-to-onset of 33 days (10). The increased reports of cases in the last years are perhaps consistent with growing recognition of this new clinical syndrome, as well as the more widespread use of ICIs. High-dose IV corticosteroids and withdrawal of ICI are considered the first-line therapy (1, 91, 92), while alemtuzumab (anti-CD52 antibody), antithymocyte globulin (anti-CD3 antibody), and abatacept (a CTLA-4 agonist) have been proposed in corticosteroid-resistant forms (Figure 1) (87, 93, 94). Retrospective data suggest that earlier (within the first 24 h) and high doses (501–1,000 mg/day) of corticosteroids lead to an improved outcome (95). Prompt diagnosis and immediate treatment of ICI-myocarditis becomes a critical issue among the cardio-oncology population, as indications for ICI increase for various cancer types. In 2021, nearly 50% of cancer patients are eligible for ICI treatment. In many cases, ICIs are combined with other cancer therapies with their own inherent cardiotoxicities (96–98). In addition, long-term cardiovascular effects of ICI become an important consideration as a growing number of cancer patients respond to therapy (99–101). Finally, the emergence of ICI-myocarditis has opened new avenues for more fundamental investigation about the role of immune checkpoints (e.g., PD-1/PD-L1 signaling) in other forms of inflammatory heart disease (102, 103). These issues need to be a focus of future investigations.

### Ventricular Arrhythmias and Myocarditis

AM can be complicated by VA. Specifically, ~40% of patients presenting with life-threatening VA can experience a recurrence at a median time of 8 months based on a recent international registry including 156 patients (104). Factors associated with arrhythmic recurrence were initial presentation with sustained VT, LGE involving ≥2 myocardial segments, and absence of T2-weighted short-tau inversion recovery (STIR) signal suggestive for residual edema on CMRI (104). In this registry, 98 patients underwent EMB showing in the large majority of cases a lymphocytic myocarditis (88.8%). An immunosuppressive therapy was initiated in 21% of cases and there was no difference in the use of immunosuppressive therapy between patients who subsequently experience an arrhythmic recurrence vs. those who did not (104). A second registry of 185 patients with VA (including VF/VT, non-sustained VT, and Lown's ≥2 premature ventricular complexes [PVC]) and myocarditis confirmed a 30% of recurrence of malignant VA at 2 years (105). Another study evaluated 58 patients with histologically proven lymphocytic myocarditis and VA as above described who underwent immunosuppressive therapy vs. a matched population of 58 cases not treated with immunosuppressive agents (2). Immunosuppressive therapy in most patients was a combination of prednisone 1 mg/kg for 6 month and AZA 2 mg/kg for 1 year. Alternatively, mycophenolate mofetil at dosage of 1–3 g/day was used instead of AZA. At 24-month follow-up, no significant differences in VF/VT occurrence were observed in patients treated with immunosuppressive agents vs. those who did not (10 vs. 17%, respectively, *p* = 0.42), even if patients who were treated with immunosuppressive agents showed a significant reduction in the PVC burden (2). Another prospective registry included 107 symptomatic patients with >5,000 PVCs/24 h without ischemic etiology who underwent a combination of laboratory testing, FDG-PET scan, CMRI and EMB (106). A positive FDG-PET scan consistent with cardiac inflammation was observed in up to 51% of patients and CS was the final diagnosis in 24% of patients with positive FDG-PET scan. Patients with signs consistent with myocarditis started an immunosuppressive therapy (prednisone 40 mg for 3 months) alone or in combination with catheter ablation, showing an optimal response in 67% of cases. Optimal response was defined as a reduction in the PVC burden >80% and negative FDG-PET scan at follow up. Furthermore, patients with LV systolic dysfunction showed an improvement in 37% of cases with a mean increase in LVEF of 13% (106). Although these studies are promising, the lack of randomization vs. a control group, the absence of reports of side effects and the fact that the immunosuppression therapy did not significantly reduce VF/VT or cardiovascular death cannot routinely support the use of corticosteroids in the management of patients with myocarditis complicated by VA or frequent PVC. Specific randomized trials are required to assess whether immunosuppression can ameliorate myocardial inflammation and reduce the risk of major VA. In addition, VA is especially a hallmark of ICI-myocarditis. In an international registry of patients with ICI-myocarditis, consisting of 147 patients, a total of 22 (15.0%) patients experienced 1 or more life-threatening ventricular arrhythmia episodes, including 16/147 (10.9%) sustained ventricular tachycardia, 4/147 (2.7%) ventricular fibrillation, and 2/147 (1.4%) torsade de pointes (107).

### COVID-19 Associated Acute Myocarditis

Cardiac injury with release of troponin has been observed quite often in patients who were hospitalized with COVID-19 (108), nevertheless cases of well-characterized AM are anecdotal (3). Data on clinically suspected AM complicated by acute HF among hospitalized patients with COVID-19 suggests a 0.12% incidence (109). Nevertheless, good data on the incidence of AM are still lacking. It has been recognized that asymptomatic forms of AM associated with severe acute respiratory syndrome coronavirus 2 (SARS-CoV-2) exposure range between 0.3 and 3% based on a CMRI diagnosis. This population has been largely studied among athletes who underwent systematic cardiac tests (ECG, troponin assessment, or transthoracic echocardiography) and, when clinically indicated, CMRI (110–112). It must be acknowledged that proportionally, individuals with mild COVID-19 related symptoms have a higher likelihood of signs of myocardial inflammation compared with asymptomatic individuals. Patients with cardiac tests consistent with AM should be advised not to practice vigorous physical activities in the 3–6 months following SARS-CoV-2 exposure if they have preserved LVEF, whereas if patients have reduced LVEF, patients should initiate specific HF therapies (113), while there is no indication for immunosuppression. Patients complaining of cardiac symptoms or signs associated with COVID-19 and diagnostic findings consistent with AM can be further divided between those with COVID-19 associated AM with concurrent pneumonia and those without pneumonia (isolated COVID-19 myocarditis). Delayed-onset AM has been described after SARS-CoV-2 exposure and typically these patients can present with high titer of SARS-CoV-2-specific antibodies and recent history consistent with COVID-19 in the absence of SARS-CoV-2 by RT-PCR on a nasopharyngeal swab. Delayed-onset myocarditis is thought to be triggered by SARS-CoV-2 induced immune-mediated reactions. Immunomodulating therapies include non-steroidal anti-inflammatory drugs (NSAIDs) to relieve chest pain, low dosage of colchicine in case of associated pericardial involvement. Corticosteroids are generally used in patients with delayed onset AM that present an associated hyperinflammatory status (114, 115). In severe COVID-19 AM presenting as FM, EMB can be deemed necessary with the aim to differentiate AM from sepsis-induced acute cardiomyopathy, especially in patients with hyperinflammatory status. Identification of inflammatory infiltrates in the myocardium could support the empirical use of immunosuppressive drugs (33), even if, diffuse inflammatory infiltrates have been rarely seen (116). Hyperinflammatory status and acute HF/cardiogenic shock in which a predominant septic state has been excluded could be treated with immunosuppressive treatments, as suggested by small series where intravenous corticosteroids have been associated with a favorable prognosis (114, 115). This condition has been termed multisystem inflammatory syndrome in adults (MIS-A) and is often associated with a delayed onset of myocarditis. The condition is usually associated with high levels of inflammatory biomarkers and ferritin (117). The Multisystem inflammatory syndrome in children (MIS-C) presents overlapping characteristics with myocarditis in adults (118). It has been that although a third of patients with MIS-C can require a t-MCS, but none died in a series of 35 children who were treated with IVIG plus a third with the addition of corticosteroids (119). Finally, patients with concurrent severe myocarditis, pneumonia, and respiratory insufficiency should receive corticosteroids (120). A review article that collected data on 38 published cases of COVID-19 associated AM reported use of corticosteroids in 34% of cases and a mortality of 15% (121), even if larger series are needed to better understand optimal therapies.

### mRNA COVID19 Vaccine-Related Acute Myocarditis

The association between vaccine administration and the onset of myocarditis is supported by several case reports, case series, and at the level of the national health care system (4–7, 122–124). The United States Vaccine Adverse Event Reporting System (VAERS), even if subject to bias, also revealed a clear signal for vaccine-associated myocarditis with nearly 1,300 cases reported from more than 350 million doses in the United States (8). Most cases have been reported in young men, thus, for 18–24-year-old males, the expected prevalence of vaccine-associated myocarditis is ~3 cases per 100,000 doses (0.003%) based on VAERS data (8). Nationwide observational data confirmed a COVID-19 vaccine-associated myocarditis at ~3 per 100,000 patients (0.003%) vs. ~11 per 100,000 patients (0.01%) for acute COVID-19 myocarditis (125). An analysis conducted in England revealed that the increased risk of myocarditis associated with the two mRNA vaccines was present only in those younger than 40 years (6).

Historically, the vaccine that is most associated with myocarditis is the anti-smallpox (10, 126). Smallpox vaccine was associated with eosinophilic myocarditis, while almost all the present cases of mRNA COVID-19 vaccine are not associated with eosinophilia. We revised 90 cases published of mRNA COVID-19 vaccine myocarditis up to the end of August 2021 (see Supplementary Tables 1, 2), and we summarized major features, and anti-inflammatory and immunomodulatory drugs used. The median age at presentation was 25 years (interquartile range 17–27), in agreement with a median age observed in VAERS (8), with a marked male prevalence (93%). Even if a higher number of BNT162b2-related myocarditis is reported, disproportionality analyses using the Bayesian information component, revealed a higher likelihood of association between mRNA 1273 and myocarditis (126). In 90% of cases, myocarditis occurs after the second dose, following a median time of 3 days between the last dose and symptoms' onset, including chest pain (observed in 96% of cases) generally preceded by fever (in 85%). All these findings suggest an immune-mediated reaction related to vaccine administration. AM is generally not severe. While electrocardiographic abnormalities are present in 77% of cases, diagnostic tools revealed only a slight reduction in the LVEF (mean value of 53%) with a pericardial effusion observed in 14% of cases. Information on anti-inflammatory/immunomodulatory therapy was available for 56 of 90 patients (62.2%). In 38 out of 56 patients, the administered drugs were reported as follows: aspirin, NSAIDs, corticosteroids, IVIG, colchicine, and anakinra. Patients who received anti-inflammatory/immunomodulatory therapy did not differ in relation with age (23 ± 9 vs. 29 ± 19 years, *p*-value 0.10) and LVEF on the first echocardiogram (53 ± 11 vs. 53 ± 13%, *p*-value 0.90). The use of immunosuppressive therapy was similar in the adult and pediatric populations (39.5 vs. 44.4%, *p*-value 0.72). Overall, NSAIDs (including aspirin) were the most used drugs (23/56 patients, 41.1%), and aspirin was used only in 3 out of 56 patients (5.4%). Corticosteroids were used in 19 of 56 patients (33.9%), IVIG in 12 patients (21.4%), colchicine in 15 patients (26.8%), and anakinra in only 2 patients. Most of the time, immunosuppressive agents were used in combination. NSAIDs were used together with the corticosteroids in 5 patients. IVIG along with corticosteroids was used in 11 patients, including 10 pediatric patients. NSAIDs along with colchicine were used in 11 of 56 patients (19.6%). Prognosis is considered favorable, with only three (3.3%) deaths reported out of 90 patients, a figure in line with the one observed in AM patients in pre-COVID19 era (40). These data are largely consistent with a series of 139 adolescents (all with age <21 years) with suspected AM within 30 days of COVID-19 vaccination (7). In fact, the male prevalence was 91%, symptoms started a median of 2 days after vaccination, and the most common symptom was chest pain (99%) (7). Again, NSAIDs were the most used drugs in 81% of cases, followed by corticosteroids (22%) and IVIG (22%), while colchicine was administered in 8% (7). No patient died or required a t-MCS.

## Eosinophilic Myocarditis

Eosinophils have widespread procoagulant effects, including the production of tissue factor (127), oxidation of phospholipids (128) (both of which activate the intrinsic pathway), the release of platelet-activating factor (129), reactive oxygen species, and eosinophil extracellular traps (130). Moreover, activated eosinophils are potent producers of vasospastic mediators (including histamine, leukotrienes C_4_ and D_4_ and prostaglandin D_2_) and are able to modulate mast cell functions (131). Lastly, the shedding of both cytotoxic granules and pro-inflammatory mediators (i.e., tumor necrosis factor [TNF]-α, IL-1 and IL-6) are contributing factors of endothelial injury and procoagulant state (132). Eosinophil-mediated toxicity can lead to protean cardiovascular manifestations, including venous thromboembolism (133), eosinophilia-related coronary vasospasm (134), thromboangiitis obliterans-like disease (135), eosinophilic coronaritis, systemic eosinophilic vasculitis (136), eosinophilic myocarditis (137), and Loeffler cardiomyopathy, a chronic inflammatory cardiomyopathy (1, 138). The natural history of eosinophil-related heart involvement involves three successive (and potentially overlapping) phases: (1) AM, due to eosinophilic infiltration of the endocardium, that can be either asymptomatic or lead to acute HF or FM (137). High troponin levels, LV systolic dysfunction, and subendocardial LGE pattern on CMRI can be observed (2, 137) a thrombotic stage characterized by the occurrence of ventricular thrombi and the risk of systemic embolism; (3) a fibrotic stage, characterized by endomyocardial fibro-thrombosis that can lead to restrictive cardiomyopathy (i.e., Loeffler cardiomyopathy) and/or atrioventricular valvular disease (139). The diagnosis of eosinophilic myocarditis is usually straightforward in the presence of hypereosinophilia, increased cardiac troponin, and CMRI consistent with subendocardial inflammation (137). EMB can be considered when the initial presentation is characterized by cardiogenic shock (1, 33), or CMRI findings are atypical (i.e., subepicardial LGE) or when absolute eosinophil counts are within the normal range (which has been reported in up to 25% of patients with biopsy-proven eosinophilic myocarditis) (137, 139). Conversely, EMB is at risk of thromboembolism if ventricular thrombi are present, and can yield false-negative findings when endomyocardial fibrosis is prominent and eosinophil infiltration has partially or completely vanished (138). Eosinophil-related heart involvement can be encountered within the full spectrum of eosinophil-associated diseases (137), including drug hypersensitivity (even in the absence of skin manifestations) (10), parasitic infections (namely toxocariasis, trichinosis, filarial infections or sarcocystosis), aspirin-exacerbated respiratory disease, eosinophilic granulomatosis with polyangiitis (EGPA, formerly Churg-Strauss syndrome), hypereosinophilic syndromes (HES) (mainly idiopathic and *FIP1L1-PDGFRA*-associated HES, formerly chronic eosinophilic leukemia) and high-grade hematological malignancies [e.g., Hodgkin and angioimmunoblastic T-cell lymphomas, as well as B-cell acute lymphoblastic lymphoma with t (5, 14) (q31;q32); IGH-IL3 rearrangement (140). In a review of 179 cases of biopsy-proven eosinophilic myocarditis, the main identified causes were drug hypersensitivity, EPGA, HES and parasitic infection, accounting for 34%, 13% and 8% of cases, respectively, while 36% of cases were idiopathic or eosinophilic myocarditis with undefined cause (137).

Heart involvement is the leading cause of death in patients with EGPA and is more frequent in antineutrophil cytoplasmic antibodies (ANCA)-negative patients (141). Of note, the differential diagnosis between ANCA-negative EGPA and HES is a frequent diagnostic and therapeutic dilemma. The European Respiratory Society and European Federation of Internal Medicine-endorsed Task Force suggested restriction in the use of EGPA to patients with eosinophilic asthma who test positive for ANCA and/or who exhibit genuine features of vasculitis (either biopsy-proven or clinical surrogates) (142). Likewise, in a retrospective analysis of 166 patients with blood eosinophilia >1,000/mm^3^ and systemic manifestations, it was recently suggested that serum CRP levels could be a reliable biomarker able to distinguish EGPA from idiopathic HES, with low (i.e., <36 mg/L) levels being suggestive of idiopathic HES rather than EGPA (143). A workup to identify associated systemic disorders should be performed in all patients with eosinophilic myocarditis. The workup should include testing for ANCA (positive in 10–40% of EGPA patients), serological testing for toxocariasis (which has a broad geographic distribution), ova and parasite tests (while further serologies for parasitic infections are generally guided by the patient's country of origin, travel history and dietary habits), serum vitamin B12 and tryptase levels (which are sensitive for the diagnosis of myeloid variant HES), total IgE levels (which are suggestive of reactive polyclonal eosinophilia mediated by IL-5, when elevated), lactate dehydrogenase (suggestive of lymphoma), thoraco-abdominopelvic CT scan (seeking for extra-cardiac eosinophil-related organ involvements as well as underlying solid or hematological malignancies). Furthermore, brain CT or brain MRI should be performed when embolic stroke is suspected in patients with eosinophilic myocarditis or Loeffler cardiomyopathy (144). Additionally, testing for *FIP1L1-PDGFRA* fusion gene should be performed in selected cases when clinical (e.g., male sex, splenomegaly), biologic (e.g., high B12 vitamin and/or tryptase levels) features and/or primary resistance to steroids are observed (145). Polymerase chain reaction testing for specific viruses (e.g., Herpesviridae, especially HHV 6) and the RegiSCAR scoring system can be useful in patients with suspected Drug Reaction with Eosinophilia and Systemic Symptoms (DRESS) (146). Additional imaging, endoscopic and histologic investigations are usually performed on a case-by-case basis after first-line investigations. In a retrospective series of 19 patients with biopsy-proven myocarditis with fulminant presentation, the rate of either cardiac death or heart transplantation at 60 days was up to 26% (11).

**Figure 3:**
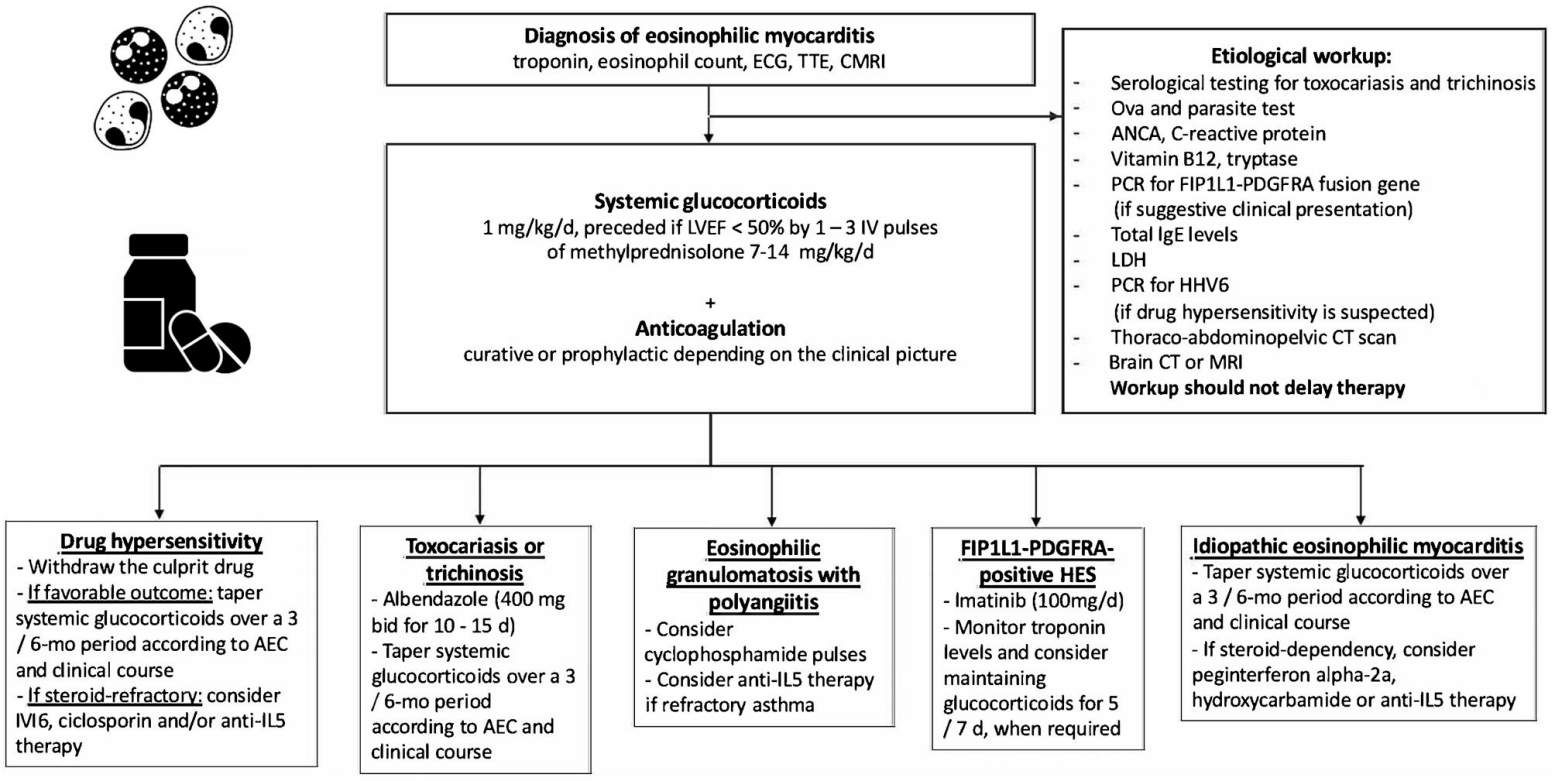
Etiological workup and immunosuppressive treatment strategies used for eosinophilic myocarditis. TTE, transthoracic echocardiography; CMRI, cardiac magnetic resonance; LVEF, left ventricular ejection fraction; ANCA, antineutrophil cytoplasmic antibodies; PCR, polymerase chain reaction; LDH, lactate dehydrogenase; HHV6, human herpes virus 6; CT, computed tomography; d, day; mo, month; AEC, absolute eosinophil count; HES, hypereosinophilic syndrome.

The cornerstone of the treatment relies on systemic glucocorticoids, starting dose: 1 mg/kg qd, preceded in case of severe LV systolic dysfunction by intravenous pulses of 7.5–15 mg/kg of methylprednisolone for 1–3 days (Figures 1, 3) (137, 139). In patients at risk of strongyloidiasis (owing to their past travel history), concomitant prescription of a single dose of ivermectin (200 μg/kg) is warranted to prevent *Strongyloides stercoralis* hyperinfection. When toxocariasis or trichinosis are evidenced, a 10/15-day course of albendazole (400 mg bid) is warranted (147). Likewise, in patients with evidence of intracavitary thrombus, anticoagulation should be initiated (while prophylactic anticoagulation is mandatory in all other patients until absolute eosinophil counts normalize). The diagnoses of myeloid variant HES, DRESS or EGPA should be suspected and investigated accordingly, after 2–4 days of corticosteroid-refractory eosinophilia. Specifically, the treatment of *FIP1L1-PDGFRA*-positive HES relies on the tyrosine kinase inhibitor imatinib (100 mg/d), and eosinophils generally plummet within days after imatinib initiation (145). Yet, transient worsening of HF after onset of imatinib has been reported, likely due to treatment-induced lysis of eosinophils (148). Conversely, IVIG and/or cyclosporine are the most common drugs used for the treatment of corticosteroid-refractory DRESS (149, 150), yet benralizumab (a humanized afucosylated monoclonal antibody that targets IL-5 receptor α) is on the rise in this setting (151). Historically, besides systemic corticosteroids, the treatment of EGPA-associated eosinophilic myocarditis complicated by severe HF relies on cyclophosphamide pulses (152, 153), yet it should be emphasized that there is no data proving that adding cyclophosphamide pulses to steroids improves outcomes. Whatever the underlying disorder, the aim is to quickly and persistently normalize eosinophil count (< 500/mm^3^). Of note, both in EGPA (154–156) and in *FIP1L1-PDGFRA*-negative HES (157, 158) targeting IL-5 has emerged as clinically relevant. Anti-IL-5 agents, such as mepolizumab and benralizumab are likely to become game changers and tend to replace the use of disease-modifying anti-rheumatic drugs (i.e., AZA, methotrexate, peginterferon alpha-2a and hydroxycarbamide), even if trials are needed. In case of persistent eosinophilia and subsequent occurrence of endomyocardial fibrosis, heart surgery with resection of fibrotic endocardium (endomyocardectomy) combined with valve repair or replacement can be considered (159). Finally, in case of refractory end-stage HF, orthotopic heart transplantation has been reported to be safe and feasible in both EGPA and HES (160, 161).

## Giant Cell Myocarditis

GCM is a rare but often fatal form of AM. The pathophysiology of GCM is thought to be a T-cell mediated autoimmune process leading to diffuse or multifocal inflammatory infiltrate, including lymphocytes with multinucleated giant cells, and definitive diagnosis requires EMB. An immune-mediated mechanism in the etiology of GCM is further supported by the fact that no nucleic acids from viruses implicated in myocarditis were detected in cardiac tissue samples from 9 patients with GCM (162).

However, the characteristic giant cells can take 1–2 weeks to appear, therefore, while EMB in the first few days of the illness may suggest myocarditis, it may render a false negative result for GCM; for this reason, EMB repetition can increase sensitivity in GCM diagnosis (163). It has been estimated to occur at a rate of 1 case per 200 patients with AM and constitutes about 10% of FM (11, 13). GCM affects men and women equally with a median age at onset between 43 and 53 years. Association with other autoimmune disorders has been observed in about 20% of cases, especially autoimmune thyroiditis and inflammatory bowel disease (59). Recent data where RNA-Sequencing (RNA-Seq) was applied to a small series of GCM cases reveals a distinct transcriptomic signature for GCM compared to other forms of myocarditis (164). Specifically, it has been observed downregulation of pathways involved in muscle contraction, ion homeostasis, and cardiac conduction, potentially explaining the typical patient presentation with acute heart failure and arrhythmias) (164).

Clinically, GCM generally presents with rapid hemodynamic deterioration (FM), VA, and at times bradyarrhythmia. The rate of death or HTx has been estimated at 81% at 3 years from the initial admission when GCM presents specifically as FM (11); whereas a 73% mortality rate at 5 years has been estimated more recently considering all GCM (165). It is characterized by the lack of spontaneous recovery on t-MCS which more commonly occurs in FM. Prolonged use of intravascular microaxial pump and VA-ECMO has been reported (166–168). Pharmacologic treatment includes multi-drug immunosuppression that typically involves combinations of anti-T-cell drugs (i.e., antithymocyte globulin, muromonab and cyclosporine) and high dose corticosteroids. No standardized protocols exist, though several regimens have been proposed in recent review articles (1, 169). Clinically relevant, immunosuppressive therapy should be initiated promptly. Treatment with anti–T-lymphocyte–based and calcineurin inhibitor therapy can lead to clinical remission in up to two-thirds of patients, in particular in those not requiring t-MCS (163, 168). The initial approach may vary based on the clinical presentation. In case of FM, antithymocyte globulin (dose raging from 1 mg/kg to 300 mg in the first 3 days) associated with pulsed high-dose corticosteroids (generally 1 g methylprednisolone per 3 days) is preferred; even if alternative protocols including alemtuzumab (an anti-CD52 antibody; at dose of 15 mg per 2 days) instead of antithymocyte globulin have been reported. Cyclosporine is then added and titrated to trough levels of 150 to 250 ng/L as maintenance therapy. There is a variable rate of LVEF recovery without transplant. Dosage of oral prednisone after the acute phase is generally 1 mg/kg in the 1st months with subsequent slow tapering over 1 year, while cyclosporine is generally maintained >2 years, with a target plasma through level of 80–100 ng/L. AZA at 1–2 mg/kg/day divided into 2 daily doses or mycophenolate mofetil (500–1,000 mg BID) can be added. In case of non-fulminant presentation a combination of mycophenolate mofetil and cyclosporine (or tacrolimus, trough levels in the first 6 months: 10–15 ng/mL) and corticosteroids can be added. Also, in cases with less severe presentation, pulsed high-dose corticosteroids are still suggested. If no recovery is obtained, HTx is an effective therapy, with similar post-transplant survival in patients with GCM as in those with other causes (170). Nevertheless, recurrence of GCM can happen in up to 25% of transplant patients, and again warrants aggressive immunosuppression which is typically sufficient for disease remission (169).

## Cardiac Sarcoidosis

**Figure 4:**
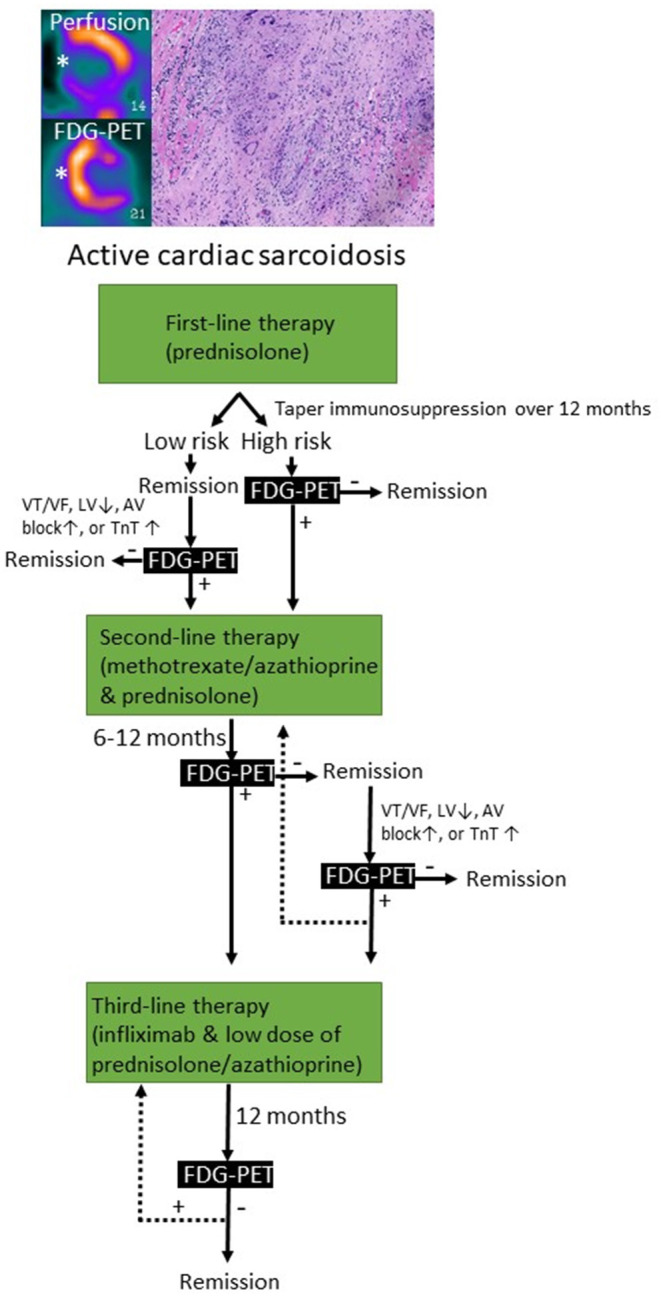
Immunosuppressive treatment strategies used for cardiac sarcoidosis based on clinical and imaging-based monitoring. FDG-PET, fluorodeoxyglucose positron emission tomography; VT/VF, ventricular tachycardia/ventricular fibrillation; LV, left ventricular ejection fraction; TnT, troponin.

CS can present as a chronic inflammatory cardiomyopathy, while infrequently can manifest as an AM (1, 165). The most reported clinical cardiac features are complete AVB, VA, LV systolic dysfunction and HF (165, 171). CS can be isolated or be part of a systemic disorder that meanly affects lungs and hilar lymph nodes. About 5% of patients with systemic sarcoidosis have clinically manifest CS (172). Myocardial histology is the gold standard of CS diagnosis but has low sensitivity (20–30%) (1). Histology is characterized by the presence of epithelioid granulomas with associated giant cells and lymphocytes, well-defined areas of inflammation and fibrosis, and absence of significant myocardial necrosis (1). Therefore, quite often the diagnosis of CS can be supported by clinical and imaging findings with contrast-enhanced CMRI and FDG-PET (1, 173). Based on this assumption, it must be accepted that if the diagnosis of CS relies on clinical and imaging criteria, we could face the risk of treating with immunosuppressive therapies patients with other inflammatory or non-inflammatory cardiomyopathies that are potentially less responsive to long-term steroid therapy or might be potentially harmed by the treatment. The immunosuppressive therapeutic approach to patients with CS is similar either presenting as a chronic inflammatory cardiomyopathy or as an AM, and it is based on corticosteroids as first line therapy (Figures 1, 4) (1). Unfortunately, no randomized controlled trial supports the immunosuppressive therapy in CS, neither for corticosteroids nor for any disease-modifying therapy. Despite lack of evidence almost all patients with CS receive systemic therapy. This is distinctly different from pulmonary sarcoidosis in which only half of the patients need systemic therapy (174). We do not know at present whether these patients with a good prognosis and mild myocardial involvement benefit from immunosuppressive therapy. Patients having at the time of initial presentation, normal LV function and only 5% of LGE, have very few adverse events (175).

### Corticosteroids

There is still controversy about the clinical efficacy, the optimal initial dose and duration of corticosteroid treatment for CS. It is plausible to assume that corticosteroids have similar effect in CS than in other forms of sarcoidosis. Consistent with this idea glucocorticoid treatment decreases myocardial troponin (176). By expert consensus, corticosteroids still constitute the first-line treatment at relatively high doses for 1–2 years. Although mechanistically plausible, we do not currently know if corticosteroid treatment improves prognosis in CS. Nevertheless, some patients do not respond to glucocorticoids. The clinical evidence for the utility of corticosteroids is based on retrospective, uncontrolled observational studies (177). Corticosteroids have been reported to improve LV systolic function at least in some patients (176, 178, 179), reverse AVB (180), and decrease VA in some studies but not in others (181, 182).

### DNA Synthesis Inhibitors

DNA synthesis inhibitors (such as AZA or methotrexate) that prevent nucleotide synthesis are used as steroid-sparing agents (183). AZA acts by suppressing the activation of Rac1 target genes such as NF-κB in T-cells (184). AZA and methotrexate have been used to enable rapid reduction in the glucocorticoid doses in order to reduce the dose-dependent side effects of glucocorticoids. Methotrexate combined with glucocorticoids decreases the risk of radiologic relapse in CS (183, 185). In pulmonary sarcoidosis, steroid-sparing agents-treated patients had a higher rate of infections compared to prednisone monotherapy (186). The major weakness of glucocorticoids and DNA synthesis inhibitors are their wide-ranging effects beyond immunosuppression.

### Infliximab or Other Anti-TNF-α Agents

TNF-α governs formation of granuloma thought NF-κB-mediated orchestration of cytokine expression and hence controls the hallmark tissue response in sarcoidosis (187). TNF-α antagonists are more selective and effective inhibitors of NF-κB activation than glucocorticoids and thus lack most of glucocorticoid side effects. However, not all the immunosuppressive effects of glucocorticoids may be mediated via NF-κB. Current recommendations based on expert consensus suggest anti-TNF-α agents to be used as a third-line therapy in the management of severe refractory sarcoidosis (188). Infliximab is a chimeric IgG1 monoclonal antibody that binds TNF-α with high affinity and neutralizes its effect in promoting inflammatory response. In pulmonary sarcoidosis, randomized, controlled trials with infliximab have shown that it is safe to use if proper precautions are followed (189, 190). Infliximab decreases inflammatory activity measured by FDG-PET and this correlated with improvement in forced vital capacity. In pulmonary sarcoidosis FDG-PET activity is predictive for treatment response in severe and refractory pulmonary sarcoidosis (190) and might add value in individualizing infliximab treatment. The effectiveness of adalimumab in pulmonary sarcoidosis was shown in a small open-label study (191). Adalimumab reduces the relapse rate as measured by FDG-PET (183). In CS, infliximab has been used successfully as a bailout therapy in glucocorticoid failures (192, 193). In addition to being more specific and potent inhibitor of granulomatous inflammation, a major benefit of TNF-α blockers is the lack of numerous side effects typical of corticosteroids. Despite TNF-α antagonists being very effective immunosuppressants, risk of serious infections is not higher than in corticosteroids (194). TNF-α is well-tolerated at dosage <10 mg/kg even in patients with HF (195). To reduce the production of neutralizing antibodies, infliximab and adalimumab are often combined with low-dose methotrexate or AZA (196).

### Ongoing Trials

The Cardiac Sarcoidosis Multi-Center Randomized Controlled Trial (CHASM CS-RCT) is a multicenter randomized controlled trial designed to compare treatment with a higher dose prednisone vs. prednisone plus methotrexate (197). The aim is to evaluate whether a low dose prednisone/methotrexate combination have similar efficacy to standard dose prednisone leading to an improvement in the quality of life, as a result of a reduced burden of side effects. Eligible subjects will have active clinically manifest CS with advanced conduction system disease, non-sustained or sustained VA, LV or right ventricular systolic dysfunction. The primary endpoint is a measure of myocardial fibrosis/scar, summed perfusion rest score on FDG-PET scan after 6 months from randomization.

## Imaging to Guide Immunosuppressive Therapy in Myocarditis and Cardiac Sarcoidosis

Echocardiography is routinely performed in patients with suspected AM to evaluate LV systolic and diastolic function and the presence of pericardial effusion. However, its role to guide therapy is limited, since it does not allow tissue characterization. CMRI has emerged as a powerful non-invasive diagnostic tool for the assessment of edema, inflammation and fibrosis (198). According to the Updated Lake Louise Criteria, AM can accurately be diagnosed if both edema and myocardial injury (necrosis or fibrosis) are demonstrated by, respectively, T2-weighted (STIR or T2-mapping) and T1-weighted imaging (T1 mapping or LGE) (198). In healed myocarditis, residual scar can be depicted by LGE (with or without elevated focal T1-values), while persistence of edema, as assessed by T2-weighted imaging, suggests active inflammation. Moreover, CMRI is the gold standard for quantification of ventricular volumes and function. In this respect, CMRI can be used to select patients who might benefit from immunosuppressive therapy, as well as to evaluate the impact of treatment on myocardial function, ongoing inflammation and scar formation. Furthermore, assessment of the disease stage of myocarditis is especially relevant for patients with myocarditis and drug-refractory VT, as recent data show a high recurrence rate post VT ablation if signs of active myocarditis are present on EMB or CMRI (199). Importantly, the Lake Louis Criteria are less accurate in detecting active myocarditis in the context of systemic immune-mediated diseases (200, 201), making CMRI less suitable to guide therapy in this setting. In sarcoidosis, the presence of LGE is a sensitive marker of cardiac involvement, but assessment of active inflammation by T2-weighted imaging is not well-validated. However, extensive LGE (>20% LV mass) is associated with a poor prognosis and absence of LV recovery after immunosuppressive therapy with corticosteroids (202). In this respect, CMRI is mainly used for diagnosis and prognostication in CS.

New advances in the field of CMRI include the enhancement of ultrasmall superparamagnetic particles of iron oxide (USPIO), which are nanoparticles that are taken up by monocytes and macrophages, to directly visualize cardiovascular inflammatory processes (203). A pre-clinical study in a rat model with experimental auto-immune myocarditis showed that USPIO-enhanced CMRI outperformed conventional CMRI regarding the detection of myocardial inflammatory cellular infiltrates (204), but the only study in humans failed to show a difference between patients with AM (*n* = 9) and healthy volunteers (*n* = 10) (205). Therefore, there is currently no role in clinical practice for USPIO-enhanced CMRI in the diagnosis or follow-up of patients with myocarditis.

**Figure 5:**
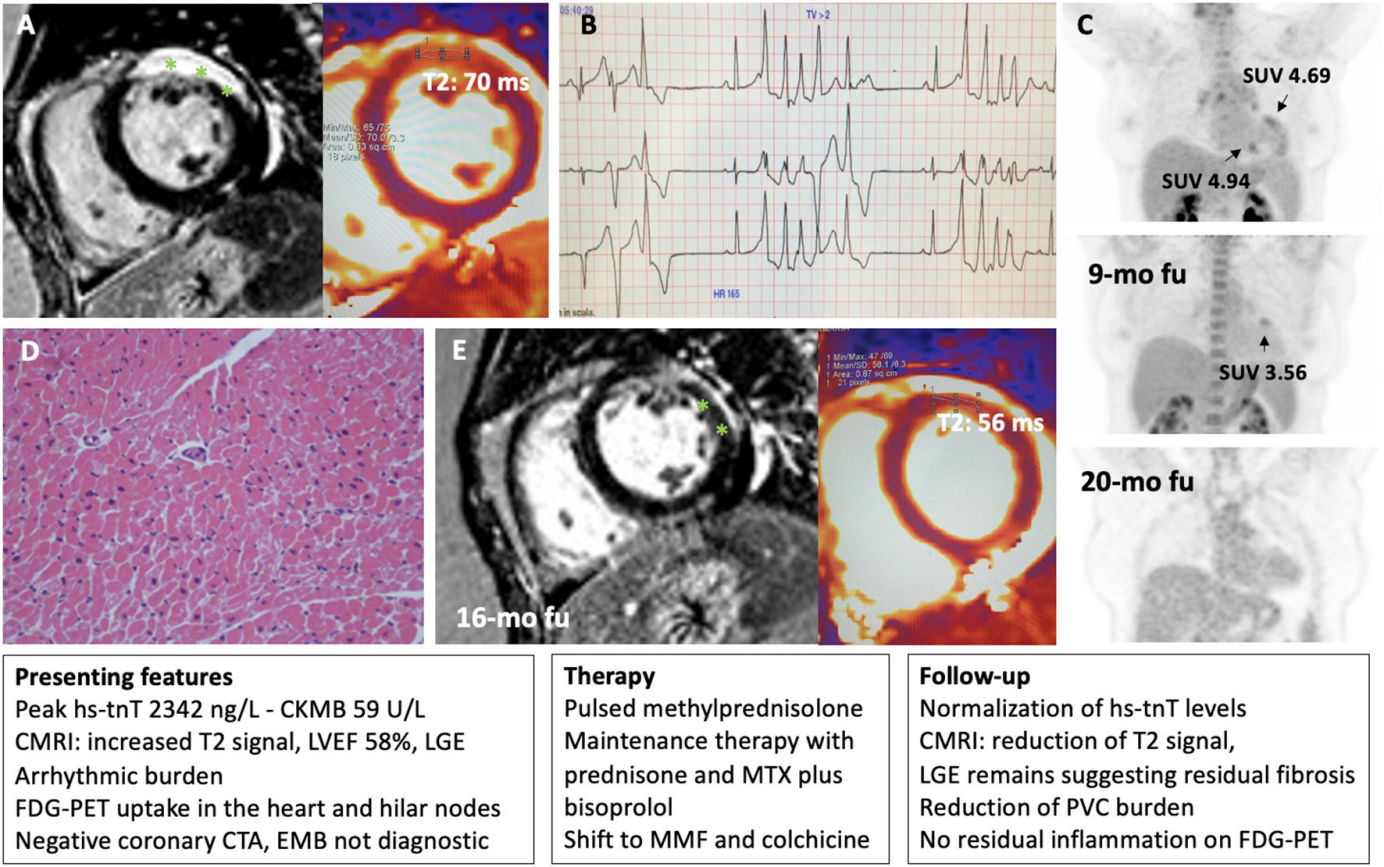
Representative patient with acute myocarditis in whom immunosuppression was guided by FDG-PET and cardiac magnetic resonance imaging (CMRI). A 49-year-old woman with a previous history of ANA positive pericarditis presented with acute myocarditis. On CMRI she presented a transmural lesion in the anterior wall as demonstrated by late gadolinium enhancement (LGE, highlighted with asterisks) and increased T2 signal (normal value <55 ms) **(A)**. Left ventricular ejection fraction (LVEF) remain preserved, but a ventricular arrhythmic burden was observed on telemetry monitoring with frequent premature ventricular complexes (PVC) and non-sustained ventricular tachycardia (NSVT) **(B)**. An FDG-PET showed 3 focal areas of uptake in the heart with increased standardized uptake values (SUV), and an uptake in hilar nodes raising the suspect for cardiac sarcoidosis **(C)**. A septal endomyocardial biopsy (EMB) from the right ventricle was non-diagnostic for myocarditis or cardiac sarcoidosis **(D)**. Peak high sensitivity troponin T (hs-tnT) levels was 2,342 ng/L. After initial pulsed methylprednisolone, prednisone was started in combination with methotrexate (MTX) and later shift to mycophenolate mofetil (MMF) and colchicine plus bisoprolol with normalization of troponin levels, and no signs of residual inflammation on FDG-PET **(C)**, and a reduction of PVC burden. Accordingly, CMRI showed a reduction of T2 signal while LGE remains suggesting an area of residual fibrosis **(E)**.

FDG-PET can detect T cells, macrophages, or granulocytes that infiltrate the myocardium, either as non-specific response to cell injury or as primary lesion in CS by an enhanced glucose metabolism after a carbohydrate-free diet. FDG-PET is recommended by several guidelines in patients with suspected active CS (172, 206), in fact, it can reveal hypermetabolic mediastinal and hilar lymph nodes differentiating CS from other autoimmune disease with cardiac involvement (e.g., vasculitis). Since FDG uptake correlates well with the level of granulomatous inflammation, it is assumed that immunosuppression should be up titrated in patients with increased metabolic activity on FDG-PET after steroid therapy has been initiated (207), while a dose reduction can be considered in patients with reduced FDG uptake. A recent study by Ning et al. (208) showed that serial FDG-PET in patients with CS altered patient management in most cases, resulting in complete weaning or significant tapering of prednisolone in 48 and 20%, respectively (Figure 4), while outcome was generally favorable. FDG-PET can be also considered as an alternative non-invasive diagnostic tool in hemodynamically stable patients with contraindication to CMRI or in patients with suspected autoimmune disease to guide immunosuppression (Figure 5) (1).

## New Insights on Pericarditis

Pathologies of the pericardium are a heterogeneous group, spanning from minimal pericardial effusion, often asymptomatic, to incessant multidrug-resistant pericarditis (209). Acute pericarditis is diagnosed based on two of the following criteria (210): chest pain, pericardial rubbing, typical changes in the electrocardiogram, with new and widespread ST elevation or PR depression in the acute phase, and pericardial effusion, which is generally mild. Increased CRP levels can support the diagnosis. The natural history of acute pericarditis can vary. In most cases, it can be self-limiting with complete resolution of the symptoms, whereas in some cases it can relapse. The development of relapses increases by up to 50% in patients who have received corticosteroid therapy for symptomatic control of the first episode. Some patients can develop incessant pericarditis, a pericarditis whose symptoms continue without interruption even for months (210). The etiology of pericarditis changes considerably depending on the geographic regions (211). In developing countries, pericarditis is often secondary to tuberculosis (212). On the other hand, in developed countries, pericarditis is more often idiopathic, secondary to autoinflammatory or autoimmune processes or following pericardial injury such radiotherapy or cardiac surgery (211).

### The Autoinflammatory Processes in Recurrent Pericarditis

Clinical and laboratory similarities between relapsing pericarditis and some autoinflammatory disorders (i.e., familial Mediterranean fever [FMF], cryopyrin-associated periodic syndromes [CAPS], TNF receptor associated periodic syndromes [TRAPS] and systemic-onset juvenile idiopathic arthritis [Still's disease]) suggested a common etiological pathway (213–224). Likely, relapsing pericarditis presents family aggregation in 10% of patients (225). FMF is an autosomal-recessive disease that mainly affects patients in the Mediterranean basin (213–218). Symptoms are characterized by self-limiting and recurrent fevers associated to serositis, affecting the pleura, peritoneum, and synovium. Although not common, pericardial effusions are found in 27% of patients with FMF, while typical chest pain is found in about 50% of pediatric patients with FMF. FMF is caused by various missense mutations of the *MEFV* gene, which encodes a pyrin that composes the NLRP3 inflammasome, NOD-like receptor family pyrin domain 3, altering its functionality. Inflammasomes play a fundamental role in innate immunity and can respond to various stimuli, including damage associated molecular patterns (DAMPs) and pathogen associated molecular patterns (PAMPs) (226, 227). DAMPs, also known as alarmins, are released from dying cells. They consist of cytosolic or nuclear-derived proteins which, in contact with the extracellular matrix, undergo denaturation processes with consequent activation of the inflammasome through interaction with pattern recognition receptors (PRRs). In this way they give rise to an inflammatory response on a non-infectious basis (termed sterile inflammation). PAMPs, on the other hand, can be identified as phylogenetically conserved molecular patterns in some microorganisms and viruses, which are recognized by toll-like receptors (TLRs), which in turn activate the inflammasome in response to an infection (227). The inflammasome is a cytosolic macromolecule composed of procaspase, ASC adapter protein and a sensor molecule containing a nucleotide-binding oligomerization domain-like receptor (NLR), which is activated by various stimuli. In FMF, functional changes in NLRP3 inflammasome cause an increased activity of the protein complex (228–230) leading to increased caspase1 activity, higher proIL-1beta into IL-1beta cleavage, and higher circulating levels of IL-1B, a master cytokine of inflammation (231). Thus, FMF manifestations are induced by increased IL-1 levels that cause a hyperactive inflammatory state.

TRAPS are autosomal dominantly inherited syndromes characterized by periodic fevers, occurring every 5–6 days for about 1–3 weeks, associated with serositis, migrating myalgia and rash, caused by missense mutation of the TNF-α receptor gene (219–222, 232). Previous studies reported an incidence of acute pericarditis in 7% of patients with TRAPS, while 25% of these patients reported chest pain with characteristics that resembles typical pericarditis pain (222). There are also oligosymptomatic forms of TRAPS, caused by mutations in TNFRSF1A, and characterized by delayed onset in which pericarditis can be the only manifestation (221). All these observations shed light on the inflammasome, and the hyperproduction of IL-1 in relapsing pericarditis. Similarly, to what observed in the above-mentioned autoinflammatory disorders, in patients with relapsing pericarditis physical injuries *via* DAMPs as well as infectious agents via PAMPs' pathways can elicit inflammasome hyperactivity and IL-1 overproduction.

### Pericarditis as an Autoimmune Process

Pericarditis can also be a complication of various autoimmune diseases, including SLE, rheumatoid arthritis (RA), Sjogren's syndrome, Behcet's disease, chronic inflammatory bowel diseases and vasculitis, including giant cell arteritis or ANCA-associated vasculitis (233). In SLE, pericarditis is common, affecting ~50% of patients, and generally occurs during disease flares. Pericarditis is usually associated with other serositis, malar rash, arthritis and leukopenia. The severity of pericarditis correlates with multiple serosal involvement. SLE therapies are normally effective (234–236). In RA, about 30% of patients have asymptomatic pericardial effusion on echocardiography, but <10% of cases develop symptomatic pericarditis. The incidence of pericarditis in RA patients is higher in those with more severe forms of RA, and higher levels of rheumatoid factor and anti-cyclic citrullinated peptide antibodies (237). Pericarditis can also be the initial sign of a new autoimmune disorder; thus, workup should be prompted after the first episode. Nevertheless, testing for antibodies in all patients with pericarditis is not recommended in the absence of signs or symptoms consistent with an autoimmune disorder (210).

### Pericarditis of Uncertain Classification (Post-cardiac Injury)

Myocardial infarction, radiotherapy, cardiac surgery or even minor procedures such as the positioning of pacemaker leads, or radiofrequency ablations can cause pericardial layers' inflammation. Oxidative stress, cell death or tissue damage can produce the release of autoantigens and, due to altered expression or post-translational modifications, these autoantigens could trigger tolerance break after epitope spreading (238). The prevalence of anti-nuclear antibodies is 43% in patients with relapsing pericarditis, while it is 10% in healthy individuals. Similarly, anti-heart antibodies and anti-intercalated disk antibodies are found in 67.5% of patients with relapsing pericarditis (210). The presence of these autoantibodies could be explained by the release of autoantigens by physical tissue injury, then the exposure of autoantigens would trigger a T/B-cell autoimmune response. Alternatively, these autoantibodies can be just an epiphenomenon. Myocardial injury can cause the release of DAMPs and the consequent activation of the inflammasome with IL-1 overproduction. This hypothesis is corroborated by good response to anti-IL-1 drugs in patients with relapsing pericarditis secondary to myocardial or pericardial mechanical injury (239).

### Pericarditis as a Systemic Disorder With Pleuro-Pulmonary Involvement

Diseases of the pericardium can be isolated or be part of a systemic condition associated a striking increase in CRP levels, erythrocyte sedimentation rate (ESR) values and neutrophilia (240–242). Approximately 53% of cases have associated pleuro-pulmonary involvement, 9% have hepatic involvement and 5% have peritoneal involvement (242). These conditions are observed more frequently in the pediatric population. Chest CT scan generally shows bilateral pleural effusion with areas of pulmonary atelectasis. Misdiagnosis with pneumonia can lead to antibiotic therapies, especially at the onset when pericardial effusion is mild. When final diagnosis of pericarditis is reached, NSAIDs (e.g., Ibuprofen 600 mg tid) and corticosteroid therapy can improve the condition. Too rapid steroid tapering can lead to pericarditis recurrence and a corticosteroid-dependent condition.

### Pericardial Effusion

Pericardial effusion can be isolated or frequently associated with an underlying pericarditis (243). The symptoms span from absent or mild to severe, especially in case of rapid formation. The pericardium tends to adapt better to slowly progressing effusions, while it tends to give compression phenomena when the effusion develops abundantly and rapidly.

Pericardial effusion can result by pericarditis, edematous syndromes including HF and kidney failure, cancer, infectious diseases (i.e., tuberculosis), serositis and autoimmune diseases, and hypothyroidism (3, 212, 244, 245), even if idiopathic pericardial effusion can often occur. A pericardial effusion is defined as chronic when it lasts for more than 3 months and severe when it exceeds 20 mm in thickness. Among 100 patients with severe (>20 mm), and chronic (>3 months) idiopathic pericardial effusion, 44 patients were asymptomatic, while 56 presented with symptoms, of these 28 presented with dyspnea; 33 patients had diabetes mellitus (246). One subset of patients was symptomatic with a higher age, more likely to be diabetic, with hypertension, chronic obstructive pulmonary disease and atrial fibrillation; whereas a second subset was generally asymptomatic, younger without significant comorbidities. After a mean follow-up of 50 months, no pathology that could explain the pericardial effusion was identified and complete regression of the effusion was observed in 39%. Adverse events were observed in 38 patients, of which 8 developed cardiac tamponade (2.2%/year). Among the 100 patients, 30 underwent pericardiocentesis, 12 underwent pericardial windowing and 3 underwent pericardiotomy. Patients who underwent some invasive procedure presented worse outcomes in terms of relapse or complications than untreated patients. This study seems to emphasize that the risk of developing cardiac tamponade is quite low and therapeutic strategies should be tailored on an individual basis based on symptoms. An echocardiographic evaluation every 3–6 months is recommended for the follow-up of these patients, while invasive techniques such as pericardiocentesis or pericardiotomy, if separated from specific symptoms, are not recommended (246). Furthermore, we recently showed that a chronic pericardial effusion is present in 37% of subjects with pectus excavatum, with the size of effusion being related to the anatomical severity of the condition, and these effusions have a good prognosis (247). Thus, in presence of chronic pericardial effusion not related to pericarditis, often with normal or near-normal serum CRP, we do not recommend any therapy, in particular, we avoid immunosuppressive therapies since there is no evidence of benefit. Low-dose corticosteroids might be considered in few selected patients on a case-by-case basis, but at present no literature deals with this topic. A study reported good efficacy and safety of intrapericardial triamcinolone in patients affected by autoreactive pericarditis with pericardial effusion (248): the use of an intrapericardial route may avoid the typical side effects of the systemic use of corticosteroids. Thus, intrapericardial use of triamcinolone remains a viable therapeutic option for patients with pericarditis and pericardial effusion. Anti-inflammatory or immunosuppressive therapies are often started because the analysis of pericardial fluid is considered suggestive of inflammation, based on the Light's criteria validated for the evaluation of pleural fluid. Data from a recent study determined the reference values of analytes and cells in pericardial fluid (249). Specifically, proteins are 1.7–4.6 g/dl, albumin 1.19–3.06 g/dl, LDH 141–2613 UI/L, total protein in pericardial fluid/serum ratio 0.29–0.83, LDH in pericardial fluid/serum ratio 0.4–42.99. According to the Light's criteria (250), pleural fluid is defined as inflammatory when at least one of the following criteria is satisfied: fluid/serum protein ratio >0.5, fluid/serum LDH ratio >0.6, and fluid LDH >2/3 of the upper limit for serum levels. The new reference values observed in this population should lead to a reappraisal concerning the classification of pericardial fluid as exudate or transudate based on Light's criteria. Efforts should be taken to stop interpreting pericardial fluid as an exudate or transudate based on evaluation tools that are not validated for this type of fluid, given the risk of misinterpreting non-inflammatory effusions into inflammatory exudates. Elevated LDH found in physiological pericardial fluid might be caused by the release of LDH by mesothelial cells, which are particularly abundant in normal pericardial fluid (249).

### COVID-19 Associated and mRNA COVID-19 Vaccine-Related Acute Pericarditis

Based on a retrospective cohort study, of 718.365 patients with COVID-19, 10.706 (1.5%) developed new-onset pericarditis. Six-month all-cause mortality was 15.5% (*n* = 816) for pericarditis and 6.7% (*n* = 356) in matched controls (*p* < 0.0001), odds ratio 2.55 (95% CI: 2.24–2.91) (251). At present, only 2 published studies focused the attention toward anti-COVID-19 vaccine-related acute pericarditis. Barda et al. reported in Israel an incidence of 26 cases out of 884.828 vaccinated individuals (3/100.000) vs. 18 out of 884.828 unvaccinated controls (2/100.000); RR 1.27 (p = non-significant) (124). Diaz et al. described 37 cases in US, with an incidence of 1.8/100.000 (252). The mean monthly number of cases of pericarditis during the prevaccine period was 49.1 (95% CI, 46.4–51.9) vs. 78.8 (95% CI, 70.3–87.9) during the vaccine period (*P* < 0.001). A total of 15 cases occurred after the first dose and 22 after the second dose; 27 out of 37 subjects were males and median age was 59 years; 13 were admitted to the hospital (median stay, 1 day), none to intensive care. No patient died.

## Therapy of Pericarditis

### NSAIDs

**Figure 6:**
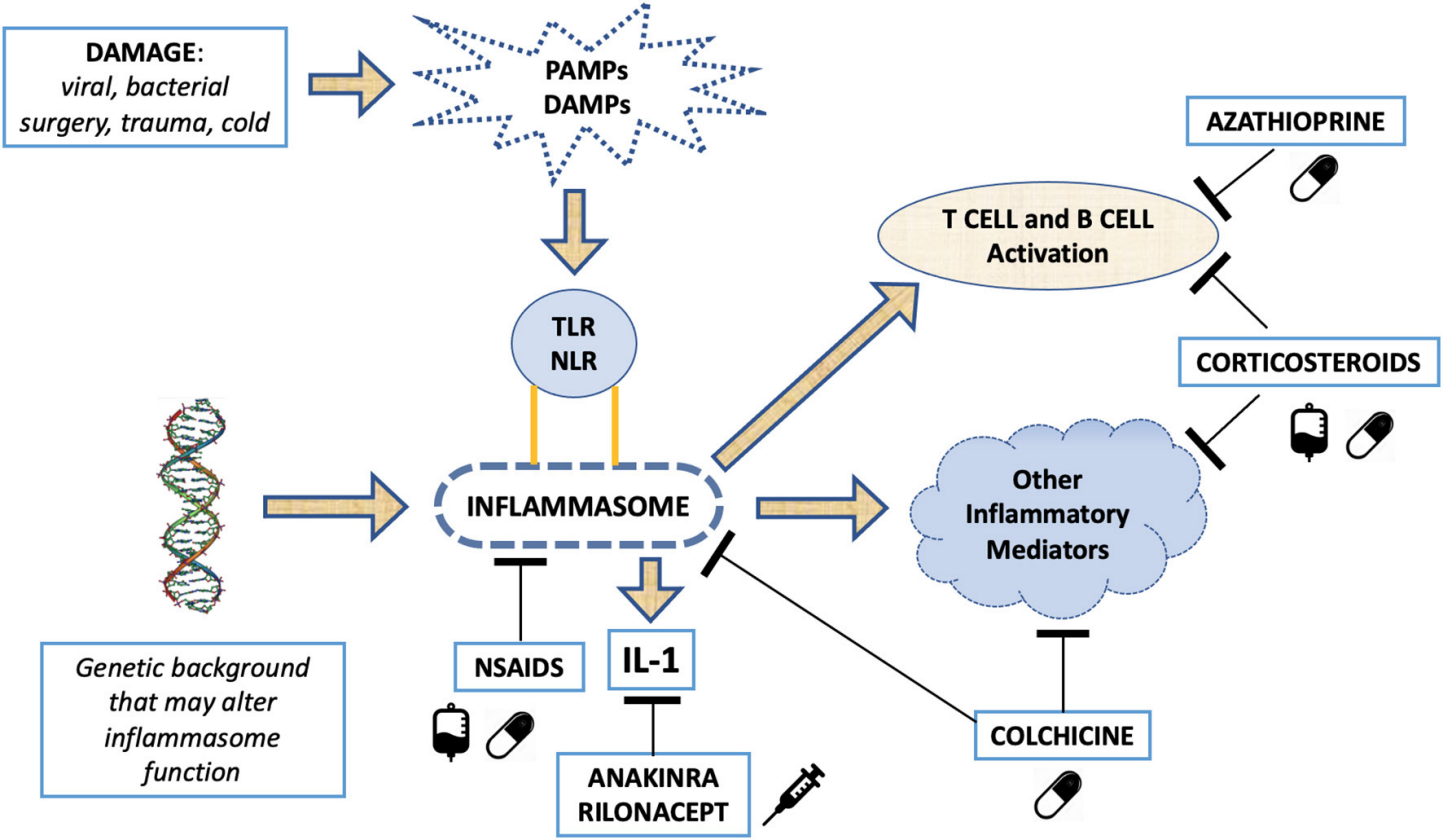
The inflammasome-mediated inflammatory cascade and the location of the effect of currently available drugs for recurrent pericarditis. Non-specific triggers such as structural damage or microbial agents may interact with specific receptors such as NLR and TLR and thus activate the inflammasome. This naturally occurs, but a genetic background may alter the inflammasome response and consequently generate a pathologic response with a sustained inflammatory state. NSAIDs perform their effect directly on inflammasome activation. Azathioprine and corticosteroids carry out their effect mainly on B and T lymphocytes. While Anakinra and Rilonacept directly inhibit IL-1 effects, both colchicine and corticosteroids perform their action on other inflammatory mediators released after inflammasome activation. Furthermore, colchicine also exerts an inhibitory effect on inflammasome activation. PAMPs, pathogen-associated molecular patterns; DAMPs, damage-associated molecular patterns; TLR, toll-like receptor; NLR, NOD-like Receptor; NSAIDs, non-steroidal antiinflammatory drugs.

**Figure 7:**
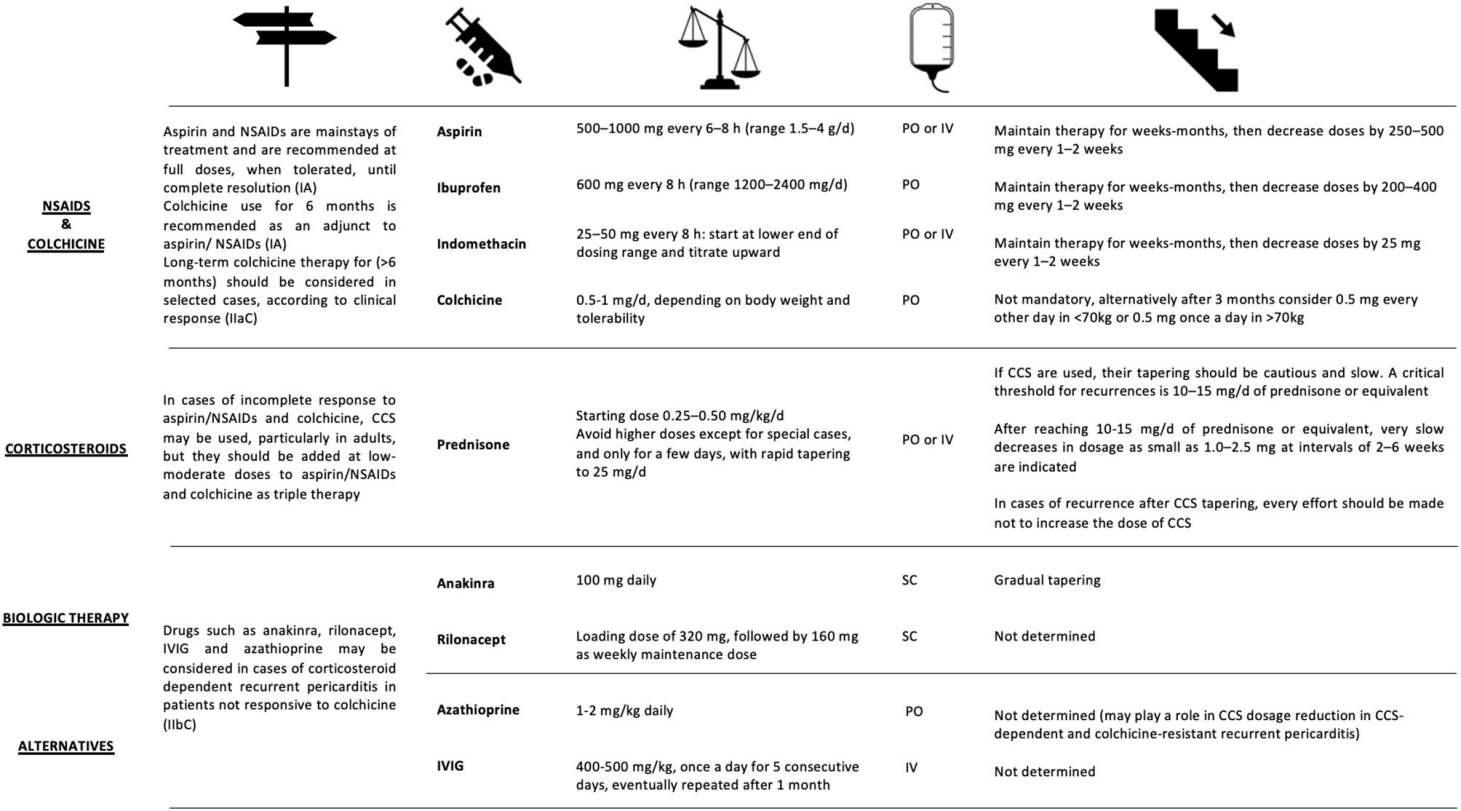
Immunosuppressive treatment strategies used for acute and recurrent pericarditis. In brackets are reported recommendation and level of evidence based on guidelines. NSAIDs, non-steroidal anti-inflammatory drugs; h, hours; d, day; PO, per os; IV, intravenous; CCS, corticosteroids; IVIG, intravenous immunoglobulin; SC, subcutaneous. Adapted from Adler et al. (210).

NSAIDs represent the first line of therapy, exerting their action both on the pathogenesis of pericarditis and on the control of symptoms. Understanding the role of inflammasome in the pathogenesis of relapsing pericarditis explains their effectiveness (Figure 6). Numerous NSAIDs are used for relapsing pericarditis therapy, including ibuprofen, indomethacin and acetylsalicylic acid (ASA). All these NSAIDs are recommended in high doses as the first line of pericarditis treatment by the European Society of Cardiology (Figure 7). The duration of treatment is variable, but in any case, prolonged (210, 253).

### Colchicine

The rationale behind the use of colchicine in pericarditis arises from the observation of good efficacy results in the control of serositis during FMF and subsequently in the control of pericarditis associated with other serositis during FMF (254). Colchicine performs its functions by inhibiting the activation of pore formation carried out by P2X2 and P2X7 receptors, that concur to the activation of inflammasome, and by inhibiting NACHT-LRRPYD-containing protein 3 inflammasome (255). The combined use of NSAIDs and colchicine has produced positive results on pericarditis in numerous clinical trials, where their use has favored both the control of symptoms and the prevention of relapses (255–261). Colchicine during relapsing pericarditis should be administered early without loading dose and its dosage might be adapted to the patient's weight: in general, we start with a dose of 0.5 mg per day and, if tolerated, the dose is then increased to 0.5 mg BID or 1 mg OD, based on compliance and tolerability. The most frequent side effects are gastrointestinal, with diarrhea that occurs mainly at the beginning of therapy in ~10%. The dosage of colchicine can possibly be reduced in patients with this type of disorder.

### Corticosteroids

The use of corticosteroids in pericarditis remains controversial. If they find their indication for the forms of pericarditis caused by autoimmune processes or in the forms resistant to the combined therapy of NSAIDs and colchicine, the probability of generating dependence for the control of symptoms is high (210). Many patients will experience a recurrence of pericarditis upon discontinuation of corticosteroid therapy, especially when corticosteroid tapering is too rapid, thus leading to a real dependence on corticosteroids and the risk of a prolonged use (253). Chronic use of corticosteroids is burdened by numerous side effects, including weight gain, osteoporosis and possible vertebral collapse, diabetes mellitus and Cushing's syndrome (262). For this reason, the use of corticosteroids should be restricted to forms of pericarditis on an autoimmune basis or in forms in which NSAIDs and colchicine have been found to be ineffective and a contraindication to the use of anti-IL-1 drugs coexists. Bisphosphonates and vitamin D should be considered when corticosteroids are started, as they are often kept as long-term maintenance therapy.

### Azathioprine

AZA is a prodrug that is converted into 6-mercaptopurine, and which exerts its action at intracellular level through the production of thioinosinic and thioguanilic acids, interfering with the production of adenine and guanine and therefore, consequently, with the production of deoxyribonucleic and ribonucleic acid. Its use in autoimmune diseases and chronic intestinal inflammatory diseases has produced good efficacy and safety data (263). During relapsing pericarditis, AZA can represent an effective therapeutic aid: it is well-tolerated and has shown good efficacy profiles especially as a corticosteroid-sparing agent (264). However, larger clinical trials on its use in relapsing pericarditis are lacking.

### Intravenous Immunoglobulins

The use of IVIG in autoimmune diseases such as autoimmune thrombocytopenic purpura, Guillain-Barré syndrome and autoimmune demyelinating polyneuropathies or in pregnant women with SLE is now well-established (265–268). IVIG carry out their function through the blocking of the Fc-gammaRIIB receptors on macrophages and in general through the blocking of the Fc receptors. IVIG are administered at a dose of 400–500 mg per kg of body weight with one intravenous administration per day for 5 consecutive days, possibly followed by another cycles of administration at 1 month. The use of IVIG during pericarditis is limited to a few case series, and it may find a rationale in autoimmune-based forms (269).

### Emerging Treatments: Anti-IL-1 Agents

The understanding of the autoinflammatory pathogenetic mechanisms, mediated by the inflammasome, in the genesis of relapsing pericarditis has shed light on IL-1 as possible therapeutic target. All drugs blocking the action of IL-1 can represent an opportunity for the control of relapsing pericarditis (270, 271). Three anti-IL-1 drugs are currently being produced, anakinra, rilonacept and canakinumab. These drugs, but especially anakinra and rilonacept, have been studied to identify their efficacy and safety profiles in patients with relapsing pericarditis.

### Anakinra

Anakinra is a short-acting IL-1 receptor antagonist for daily subcutaneous administration with doses of 100 mg qd. It was approved in 2001 by the Food and Drug Administration (FDA) for the treatment of RA and juvenile idiopathic arthritis. Anakinra does not require dosage adjustments for the patient's age, gender or body mass index, while dosage adjustments are recommended for patients with renal impairment with a glomerular filtration rate (GFR) <50 ml /min^*^1.73 m^2^ (272). The most common side effect is the formation of reddish, slightly burning and itchy skin plaque at the injection site (273). These cutaneous lesions tend to form mainly in the 1st months of therapy and can be reverted by the use of local ice or topical application of corticosteroids. Transient and mild increases in transaminase levels or leukopenia can also occur. Latent tuberculosis reactivation has been reported during the use of anakinra, leading to screening test for latent tuberculosis before starting anakinra (274). In addition, anakinra is contraindicated in patients with hypersensitivity to *E. coli* derived proteins. The use of anakinra in cardiovascular diseases is currently under investigation in myocardial infarction, HF, AM and pericardial disease (45). In the AIRTRIP study (271), the efficacy of anakinra was tested in a randomized, double-blind placebo-controlled trial in 21 patients with relapsing pericarditis who were colchicine-resistant and corticosteroid-dependent. In the group of patients taking anakinra, only 18% experienced a recurrence vs. 90% in patients in the placebo arm. This effect also occurred in patients with relapsing pericarditis secondary to post cardiac injury pericarditis. During the AIRTRIP study, only mild adverse events were observed. In the IRAP (International Registry of Anakinra for Pericarditis) registry, the efficacy of anakinra in reducing the dose of corticosteroids was also demonstrated in patients affected by relapsing pericarditis that was colchicine-resistant and corticosteroid-dependent for symptoms control, with a reduction of the percentage of patients needing corticosteroids for symptoms control from 80 to 27% (*p* < 0.001) (275). Tapering of anakinra in relapsing pericarditis should be very slow, as new disease flares have been reported in patients who abruptly stopped the drug and in patients who discontinued its use in <3 months (275).

### Rilonacept

Rilonacept is a dimeric fusion protein formed by ligand-binding domains of IL-1R and the accessory IL-1 receptor protein linked to FC portion of human IgG1. It exerts its actions by blocking both IL-1 α and IL-1B (276). FDA approved its use in CAPS, and recently also in relapsing pericarditis (277, 278). The RHAPSODY study tested its use in patients with relapsing pericarditis associated with high CRP levels. RHAPSODY is a multicentric, double-blind, randomized trial in 86 patients with relapsing pericarditis, diagnosed based on the 2015 ESC criteria during at least a second relapse despite NSAIDs, colchicine and corticosteroids treatment or any combination of these three drugs (279). Starting dose was 320 mg, followed by weekly doses of 160 mg for 12 weeks of run-in period. All other drugs to prevent relapse were discontinued. All patients achieving a clinical response were then double-blind randomized to continue rilonacept therapy or starting placebo. Only 7% of patients experienced a new flare of disease in the rilonacept arm, while 74% of patients in the placebo arm had a pericarditis recurrence. Adverse events were reported in 74 out of 86 patients and were all categorized as mild to moderate, with mainly injection site reactions and mild upper respiratory ways infections. In four patients, adverse events led to discontinuation of therapy. Based on the results of the RHAPSODY trial (279), FDA approved rilonacept for the treatment of recurrent pericarditis in March 2021.

### Canakinumab

Canakinumab is a human monoclonal antibody directed against IL-1B, which compared to anakinra has a much longer half-life, i.e., about 22–26 days, allowing an administration every 4–8 weeks (150 mg by subcutaneous injection in adults) (280, 281). Canakinumab is approved for FMF, CAPS, TRAPS, systemic-onset idiopathic juvenile arthritis and gouty arthritis (282–288). Data regarding its use in relapsing pericarditis are limited. Canakinumab was used in a case series where the use of anakinra was avoid due to adverse reactions, and data from this study were encouraging (289), but further evidences seemed contradictory (290). Canakinumab only blocks IL-1 B, while anakinra and rilonacept block both IL-1 α and IL-1B, probably explaining the better results of the latter.

### Candidates for Anti-IL-1 Agents

It is important to identify the right candidate for anakinra and rilonacept (291, 292). Patients with an overt inflammatory phenotype, suffering from pericarditis with pleuropulmonary involvement, with elevated CRP levels, fever, neutrophilic leukocytosis, with repeated hospitalizations for pericarditis, are the best candidates for anti-IL-1 therapy. Prior to the administration of anti-IL-1 drugs, guideline-driven therapy should be administered, with the use of a combination of NSAIDs and colchicine. Anti-IL-1 agents could be considered before corticosteroids, and this is particularly true for pediatric patients. Also, anakinra and rilonacept may be used in patients where use of NSAIDs or corticosteroids is contraindicated, such as anticoagulated patients, patients with renal failure, gastrointestinal hemorrhages, ischemic heart disease or recent cardiac surgery. On the contrary, their use is contraindicated in pericardial effusion or in aspecific/atypical presentations of chest pain with normal serum levels of CRP.

## Imaging to Guide Immunosuppressive Therapy in Pericarditis

Echocardiography is the first-line imaging tool when acute pericarditis is suspected. Although no abnormalities are seen in around 40% of cases, the presence of new or worsening pericardial effusion is considered diagnostic (293, 294). In uncomplicated cases with no or a small effusion, further imaging is usually not required. In case a moderate or large pericardial effusion is present, its hemodynamic consequences can be assessed with Doppler echocardiography. Echocardiography is also the main tool to guide pericardiocentesis in case of tamponade, and to evaluate residual effusion during follow-up.

**Figure 8:**
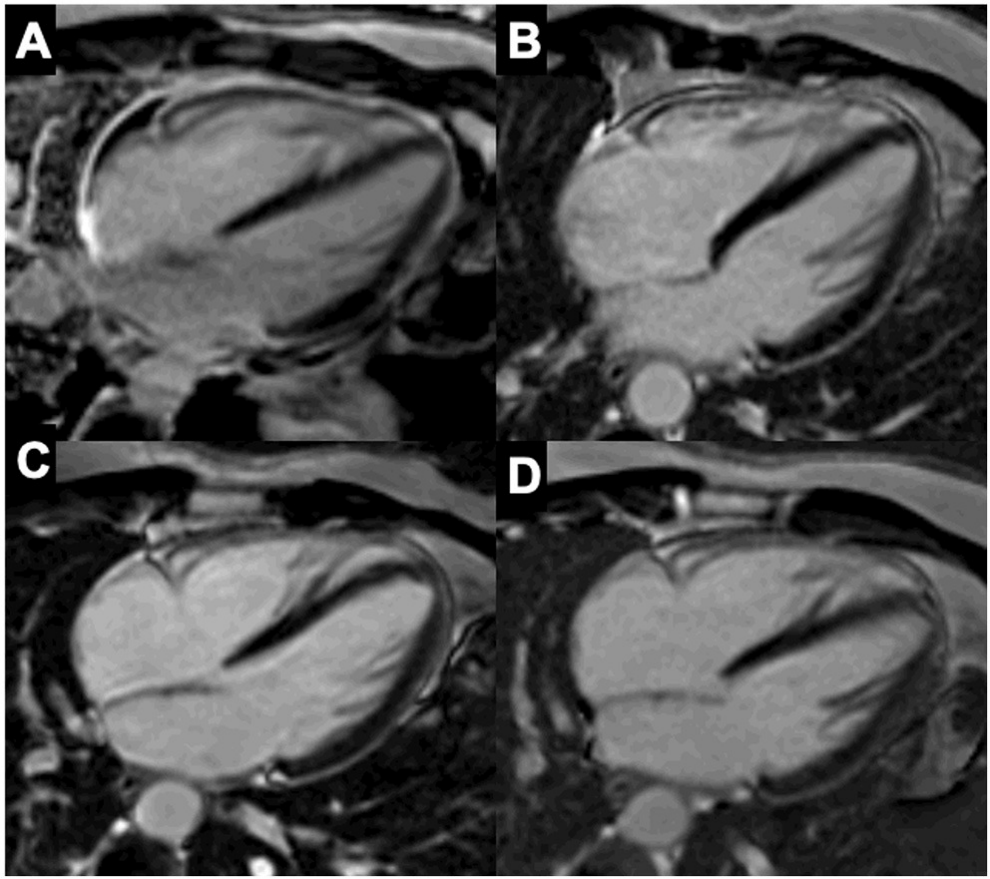
Representative patient with pericarditis and resolution of the pericardial inflammation on sequential CMRI scans. Four-chamber late gadolinium enhancement (LGE) PSIR cardiac MRI images of a 35-year-old male patient who presented with recurrent idiopathic pericarditis under NSAIDs and colchicine. **(A)** At presentation, there was diffuse pericardial thickening and LGE, and presence of pericardial effusion. High-dose corticosteroids (prednisolone 40 mg once daily) with taper schedule and azathioprine were initiated. **(B)** Re-evaluation after 3 months showed regression of pericardial LGE and disappearance of pericardial effusion. This allowed to further decrease the dose of steroids. **(C)** Follow-up cardiac MRI 5-months later showed near resolution of pericardial LGE. A low dose of steroids (prednisolone 4 mg once daily) was maintained. **(D)** Control after 1 year showed a normal pericardium without LGE. Image courtesy of Bernard Paelinck (Antwerp University Hospital).

Other imaging modalities can be useful in patients with acute pericarditis and poor echocardiographic image quality in specific settings (e.g., complicated course, large effusions), or in dubious cases, e.g., with normal CRP. On CMRI, pericardial thickening (>3 mm) and LGE, which reflects increased vascularity, are both sensitive and specific signs of active pericarditis (295). Pericardial edema can be assessed by T2-weighted STIR imaging on CMRI but might be difficult to distinguish from pericardial effusion (296). In complicated cases with relapsing pericarditis, CMRI is not only a useful tool to assess constrictive physiology and ongoing pericardial inflammation, but also to guide treatment. In a study by Feng et al. (297), it was shown that constrictive pericarditis can be reversible after anti-inflammatory therapy (NSAIDs, colchicine and/or steroids) if pericardial LGE is present on cardiac MRI. Close follow-up to evaluate improvement of hemodynamics, and pericardial effusion if present, under medical treatment by echocardiography or CMRI is recommended (293). Moreover, CMRI using T2-weighted STIR and LGE sequences can monitor the degree of pericardial inflammation (Figure 8), hereby providing important information for the clinician before anti-inflammatory or immunosuppressive medication is tapered off (296). Cardiac CT can be used to detect pericardial effusion and pericardial inflammation after contrast but implies ionizing radiation. In chronic forms of constrictive pericarditis, cardiac CT is particularly useful to assess pericardial calcifications for planning of a pericardiotomy. FDG-PET has been used to visualize pericardial inflammation and metabolic activity (298), but it is seldom performed in clinical practice and is not recommended by current guidelines (293). Nevertheless, a small study in 16 patients showed that a high FDG uptake (SUV_max_>3.0) predicted reversibility of constrictive pericarditis with steroid treatment (299). In this respect, FDG-PET may be useful in patients with non-CMRI-conditional devices to guide immunosuppressive therapy, but further studies are needed to evaluate whether it provides incremental value to CMRI.

## Conclusions

While therapies for patients with acute and recurrent pericarditis are mainly evidence-based, almost no trials are available for AM, thus immunosuppression in this setting is generally based on expert consensus. Thus, an impelling need of clinical research is to evaluate which immunosuppressive agent can be effective to improve the outcome of patients with AM and the characteristics of patients who can benefit more by immunosuppression. Thus, well-powered multicenter randomized trials are needed to test this hypothesis. In parallel, large prospective registries can better define the main determinants of outcome, even if large retrospective studies consistently demonstrated that presentation of AM complicated by reduced LVEF, HF, VA, AVB, or cardiogenic shock are associated with poor outcome (11, 40).

## Author Contributions

EA, EB, GV, MG, CV, JL, MP, AC, CP, LT, JM, and AB wrote the draft. EA, EB, GV, MG, JM, and AB revised the manuscript. All authors contributed to the article and approved the submitted version.

## Conflict of Interest

EA received a grant from the Italian Ministry of Health (GR-2019-12368506). AB: Institution received funding from Kiniksa Pharmaceuticals, Ltd., as an investigative site; unrestricted research grant from SOBI and ACARPIA; travel and accommodation for advisory committee from SOBI and Kiniksa. JM has served on advisory boards for Bristol Myers Squibb, Pfizer, Takeda, Audentes, Deciphera, Janssen, ImmunoCore, Myovant, Cytokinetics, AstraZeneca, ProteinQure, and Pharmacyclics. JM was supported by the National Institutes of Health (R01HL141466, R01HL155990, and R01HL156021). MG received consulting fees from Astrazeneca and GlaxoSmithKline. The remaining authors declare that the research was conducted in the absence of any commercial or financial relationships that could be construed as a potential conflict of interest.

## Publisher's Note

All claims expressed in this article are solely those of the authors and do not necessarily represent those of their affiliated organizations, or those of the publisher, the editors and the reviewers. Any product that may be evaluated in this article, or claim that may be made by its manufacturer, is not guaranteed or endorsed by the publisher.
