# Supplementary material for: Immunomodulating Therapies in Acute Myocarditis and Recurrent/Acute Pericarditis
Source: Front Med (Lausanne). 2022 Mar 7;9:838564. doi: 10.3389/fmed.2022.838564 (PMC8958011; doi:10.3389/fmed.2022.838564)
Supplement: Supplementary file 1 [file Table_1.pdf]

SUPPLEMENTAL TABLE 1. Case reports and cases series of published mRNA COVID-19 Related acute myocarditis up to mid-August 2021

Authorship	Journal	Publication date	Type of study	N° patients	Age	Male Sex	Type of vaccine	Dose	Days after last dose	Clinical outcome
**Ammirati E, et al.**	Int J Cardiol Heart Vasc.	31/03/2021	Case report	1	56	M	BNT162b2	1st and 2nd	3	Alive
**García JB, et al.**	Rev Esp Cardiol.	27/04/2021	Case report	1	39	M	BNT162b2	1st	21	Alive
**Albert E, et al.**	Radiol Case Rep	18/05/2021	Case report	1	24	M	mRNA-1273	1st and 2nd	4	Alive
**D'Angelo T, et al.**	Can J Cardiol.	09/06/2021	Case report	1	30	M	BNT162b2	1st	21	Alive
**McLean K, et al.**	Acad Emerg Med.	16/06/2021	Case report	1	16	M	BNT162b2	1st and 2nd	3	Alive
**Kim HW, et al.**	JAMA Cardiol.	29/06/2021	Case series	4	30	3 / 4	mRNA-1273 50% ; BNT162b2 50%	1st and 2nd	2,5	Alive (4 / 4)
**Montgomery , et al**	JAMA Cardiol.	29/06/2021	Case series	24	25	24 / 24	BNT162b2 29,2 % ; mRNA-1273 70,8 %	1st and 2nd	2	Alive (24 / 24)
**Abu Mouch S, et al.**	Vaccine	29/06/2021	Case series	6	22	6 / 6	BNT162b2 100%	1st and 2nd 83,3%	5	Alive (6 / 6)
**Cereda A, et al.**	Anatol J Cardiol.	09/07/2021	Case report	1	21	M	BNT162b2	1st and 2nd	3	Alive
**Starekova J, et al.**	Radiology	20/07/2021	Case series	5	21	4/5	BNT162b2 60% ; mRNA-1273 40%	1st and 2nd	3	Alive (5 / 5)
**Cimaglia P, et al.**	Rev Port Cardiol.	24/07/2021	Case report	1	24	M	BNT162b2	1st and 2nd	5	Alive
**Nassar M, et al.**	Diabetes Metab Syndr.	28/07/2021	Case report	1	70	F	Ad26.COV2.S	-	2	Deceased
**Marshall M, et al.**	Pediatrics	01/08/2021	Case series	7	17	7 / 7	BNT162b2 100%	1st and 2nd	3	Alive (7 / 7)
**Dionne A, et al.**	JAMA Cardiol.	10/08/2021	Case series	15	15	14 / 15	BNT162b2 100%	1st and 2nd	3	Alive (15 / 15)
**Larson KF, et al.**	Circulation	10/08/2021	Case series	6	28	6 / 6	mRNA-1273 50% ; BNT162b2 50%	1st and 2nd 83,3%	2,5	Alive (6 / 6)
**Muthukumar A, et al.**	Circulation	10/08/2021	Case report	1	52	M	mRNA-1273	1st and 2nd	3	Alive
**Rosner CM, et al.**	Circulation	10/08/2021	Case series	7	24	7 / 7	BNT162b2 71,4% ; mRNA-1273 14,3%; Ad26.COV2.S 14,3%	1st and 2nd 80%	3	Alive (7 / 7)
**Abbate A, et al.**	Int J Cardiol.	12/08/2021	Case series	2	30	1 / 2	BNT162b2 100%	1st and 2nd 50%	5	Alive (1 / 2)
**Verma AK, et al.**	N Engl J Med.	12/08/2021	Case series	2	44	1 / 2	mRNA-1273 50% ; BNT162b2 50%	1st and 2nd 50%	12	Alive (1 / 2)
**Sulemankhil I, et al.**	Cardiovasc Revasc Med.	12/08/2021	Case report	1	33	M	Ad26.COV2.S	-	2	Alive
