# Supplementary material for: Immunomodulating Therapies in Acute Myocarditis and Recurrent/Acute Pericarditis
Source: Front Med (Lausanne). 2022 Mar 7;9:838564. doi: 10.3389/fmed.2022.838564 (PMC8958011; doi:10.3389/fmed.2022.838564)
Supplement: Supplementary file 2 [file Table_2.pdf]

SUPPLEMENTAL TABLE 2. Characteristics and Therapies of published cases of mRNA COVID-19 Related acute myocarditis up to mid-August 2021

Authorship	Fever	Chest pain	Normal EKG	ST Elevation	Pericardial effusion	Lowest ejection fraction	CMR	LGE epicardial pattern	Immunomodulatory treatment	Vasopressors / Intubation / ECMO
Ammirati E, et al.	Yes	Yes	0	Yes	No	60	Yes	Yes	No	No
García JB, et al.	Yes	Yes	0	Yes	No	60	Yes	Yes	No	No
Albert E, et al.	Yes	Yes	1	No	No	65	Yes	Yes	No	No
D'Angelo T, et al.	Yes	Yes	0	Yes	Yes	60	Yes	Yes	Yes	No
McLean K, et al.	Yes	Yes	0	Yes	No	61	Yes	NA	Yes	No
Kim HW, et al.	3/4	1/4	0	4/4	4/4	55	4/4	4/4	3/4	No
Montgomery , et al	24/24	24/24	4/24	0/24	0/24	55	24/24	NA	NA	No
Abu Mouch S, et al.	6/6	6/6	0/6	5/6	0/6	55	6/6	6/6	6/6	No
Cereda A, et al.	Yes	Yes	0	Yes	No	50	Yes	Yes	Yes	No
Starekova J, et al.	3/5	5/5	NA	2/5	3/5	54	5/5	5/5	0/5	No
Cimaglia P, et al.	Yes	Yes	0	Yes	No	45	Yes	Yes	Yes	No
Nassar M, et al.	Yes	No	0	No	No	10	No	NA	No	Yes
Marshall M, et al.	5/7	7/7	0/7	6/7	0/7	60	7/7	4/7	7/7	No
Dionne A, et al.	15/15	15/15	6/15	9/15	0/15	58	15/15	NA	7/15	No
Larson KF, et al.	4/6	6/6	1/6	5/6	2/6	50	6/6	2/6	4/6	No
Muthukumar A, et al.	Yes	Yes	0	No	No	60	Yes	Yes	No	No
Rosner CM, et al.	2/6	6/6	2/6	3/6	0/6	50	6/6	6/6	3/6	No
Abbate A, et al.	2/2	2/2	0/2	2/2	2/2	17,5	1/2	1/2	2/2	Yes
Verma AK, et al.	2/2	2/2	0/2	1/2	0/2	15	1/2	NA	2/2	1/2
Sulemankhil I, et al.	No	Yes	1	No	No	60	Yes	Yes	No	No
